# Tumour resistance in induced pluripotent stem cells derived from naked mole-rats

**DOI:** 10.1038/ncomms11471

**Published:** 2016-05-10

**Authors:** Shingo Miyawaki, Yoshimi Kawamura, Yuki Oiwa, Atsushi Shimizu, Tsuyoshi Hachiya, Hidemasa Bono, Ikuko Koya, Yohei Okada, Tokuhiro Kimura, Yoshihiro Tsuchiya, Sadafumi Suzuki, Nobuyuki Onishi, Naoko Kuzumaki, Yumi Matsuzaki, Minoru Narita, Eiji Ikeda, Kazuo Okanoya, Ken-ichiro Seino, Hideyuki Saya, Hideyuki Okano, Kyoko Miura

**Affiliations:** 1 Biomedical Animal Research Laboratory, Institute for Genetic Medicine, Hokkaido University, Hokkaido 060-0815, Japan; 2 Department of Physiology, Keio University School of Medicine, Tokyo 160-8582, Japan; 3 Division of Immunobiology, Institute for Genetic Medicine, Hokkaido University, Hokkaido 060-0815, Japan; 4 Division of Biomedical Information Analysis, Iwate Tohoku Medical Megabank Organization, Disaster Reconstruction Center, Iwate Medical University, Iwate 028-3694, Japan; 5 Database Center for Life Science, Research Organization of Information and Systems, Mishima 411-8540, Japan; 6 Department of Neurology, Aichi Medical University School of Medicine, Aichi 480-1195, Japan; 7 Department of Pathology, Yamaguchi University Graduate School of Medicine, Yamaguchi 755-8505, Japan; 8 Department of Pharmacology, Hoshi University School of Pharmacy and Pharmaceutical Sciences, Tokyo 142-8501, Japan; 9 Division of Gene Regulation, Institute for Advanced Medical Research, Keio University School of Medicine, Tokyo 160-8582, Japan; 10 Department of Life Science, Shimane University Faculty of Medicine, Shimane 693-8501, Japan; 11 Graduate School of Arts and Science, The University of Tokyo, Tokyo 153-8902, Japan; 12 PRESTO, Japan Science and Technology Agency, Saitama 332-0012, Japan

## Abstract

The naked mole-rat (NMR, *Heterocephalus glaber*), which is the longest-lived rodent species, exhibits extraordinary resistance to cancer. Here we report that NMR somatic cells exhibit a unique tumour-suppressor response to reprogramming induction. In this study, we generate NMR-induced pluripotent stem cells (NMR-iPSCs) and find that NMR-iPSCs do not exhibit teratoma-forming tumorigenicity due to the species-specific activation of tumour-suppressor *alternative reading frame* (*ARF*) and a disruption mutation of the oncogene *ES cell-expressed Ras* (*ERAS*). The forced expression of *Arf* in mouse iPSCs markedly reduces tumorigenicity. Furthermore, we identify an NMR-specific tumour-suppression phenotype—ARF suppression-induced senescence (ASIS)—that may protect iPSCs and somatic cells from ARF suppression and, as a consequence, tumorigenicity. Thus, NMR-specific *ARF* regulation and the disruption of *ERAS* regulate tumour resistance in NMR-iPSCs. Our findings obtained from studies of NMR-iPSCs provide new insight into the mechanisms of tumorigenicity in iPSCs and cancer resistance in the NMR.

## Abstract (web summary)

The naked mole-rat exhibits an exceptional resistance to cancer. Here, the authors show that induced pluripotent stem cells derived from the naked mole-rat lack teratoma-forming tumorigenicity due to a naked mole-rat-specific ARF-dependent tumour-suppression mechanism.

## Graphical abstract

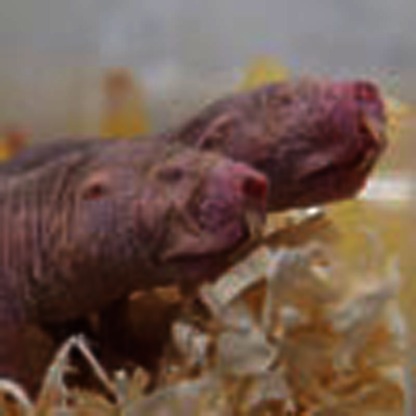

The naked mole-rat (*Heterocephalus glaber*, NMR; Fig. 1a), a eusocial subterranean mammal native to Africa^1^, is the longest-lived rodent (maximum lifespan, 30 years); its body mass, however, is similar to that of the house mouse (*Mus musculus*, Ms)^2^,^3^. NMRs exhibit extraordinary resistance to cancer, which has almost never been detected in long-term observations of captured NMR colonies^2^,^4^. In this study, we report that NMR somatic cells exhibit a tumour-suppressor response to reprogramming induction.

Cancer development and cellular reprogramming share several characteristics, including changes in global gene expression, epigenetic modification and metabolism^5^,^6^. Induced pluripotent stem cells (iPSCs) reprogrammed from somatic cells acquire pluripotency but also exhibit tumorigenicity similar to that of embryonic stem cells (ESCs)^7^,^8^, which form teratomas *in vivo*, representing a major risk factor confronting potential clinical applications^9^,^10^. The expression of many tumour suppressors, oncogenes and pluripotency genes, including *Oct4, Sox2, Klf4* and *c-Myc* (OSKM), contributes to both reprogramming and oncogenesis^5^,^11^, and the transient *in vivo* expression of OSKM has been shown to induce tumours in certain tissues^12^. These observations raise the question of whether somatic cell reprogramming conferring pluripotency and tumorigenicity can be induced in cancer-resistant animals, such as the naked mole-rat.

In this study, we generate NMR-iPSCs and show that they do not exhibit teratoma-forming tumorigenicity. Furthermore, we demonstrate that this phenotype is the result of the activation of a tumour-suppressor *alternative reading frame* (*ARF*), which is strongly suppressed in Ms-iPSCs^13^,^14^,^15^, and a unique frameshift mutation in *ES cell-expressed Ras* (*ERAS*), which positively regulates the tumorigenicity of Ms-ESCs^16^. Moreover, we identify a mechanism that we have termed ‘ARF suppression-induced senescence' (ASIS), which appears to be a NMR-specific tumour-suppression mechanism. This study provides novel insights into NMR cancer resistance and methods for generating safe iPSCs.

## Results

### Generation of NMR-iPSCs

**Figure 1:**
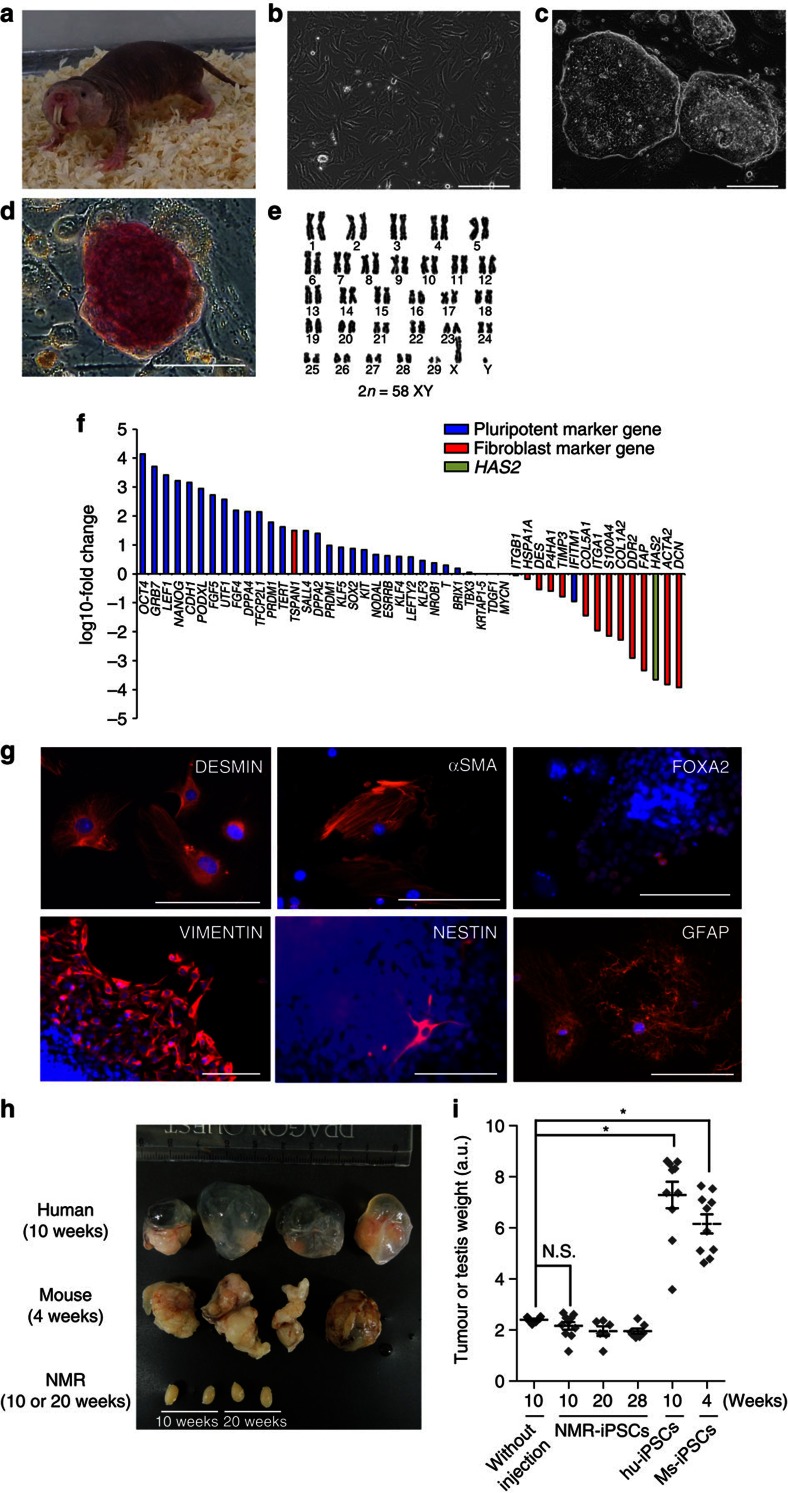
Generation of NMR-iPSCs from adult fibroblasts. (**a**) Adult NMR. (**b**) Morphology of NMR-fibroblasts. (**c**) NMR-iPSCs (clone 27). (**d**) AP activity. (**e**) Karyotype of NMR-iPSCs (clone 24) at passage 10. (**f**) RNA-seq of expression levels of selected pluripotency and fibroblast markers in NMR-iPSCs and NMR-fibroblasts. *Y* axis: ratio of the average value of fragments per kilobase of transcript per million mapped reads (FPKM) of four NMR-iPSC clones to the average of NMR-fibroblast lines. (**g**) Immunocytochemical analyses of the expression of differentiated EBs is shown as follows: mesoderm (DESMIN, α-smooth muscle actin (αSMA)), endoderm (FOXA2 and VIMENTIN) and ectoderm (NESTIN and GFAP) markers. (**h**) Tumours or testes after transplantation of human-iPSCs (10 weeks), Ms-iPSCs (4 weeks) or NMR-iPSCs (10 or 20 weeks) into the testes of NOD/SCID mice. (**i**) Weights of tumours and testes. Ten weeks (*n*=20), 20 weeks (*n*=16) or 28 weeks (*n*=10) for NMR; 10 weeks (*n*=8) for human, 4 weeks (*n*=8) for mouse. *n*: transplanted testes. *Y* axis: weights in 6+log_2_ arbitrary units. The data are represented as mean±s.e.m. *P<0.05; NS, not significant; Kruskal–Wallis test followed by the Dunn's test. Scale bar, 200 μm.

To induce reprogramming in NMR, we transduced adult skin fibroblasts (NMR-fibroblasts) using retroviral vectors expressing Ms OSKM^8^ (Fig. 1b, Supplementary Fig. 1a). Within 2–3 weeks, ESC-like colonies appeared in standard human-ESC medium containing basic fibroblast growth factor. The alkaline-phosphatase (AP)-positive colony-formation efficiency of these cells was 0.0029±0.00028% (Supplementary Fig. 1b). ESC-like colonies were expanded and designated NMR-iPSCs (Fig. 1c and Supplementary Fig. 1c). These colonies did not form under hypoxic conditions or without the introduction of c-Myc (Supplementary Fig. 1b). Although small AP-positive colonies appeared in Ms-ESC medium containing either leukaemia inhibitory factor (LIF) or LIF/2i (GSK3β and MEK inhibitors)^17^, the colonies did not expand after picking up (Supplementary Fig. 1b).

### NMR-iPSCs exhibit pluripotency but not tumorigenicity

Two NMR-iPSC clones were normal karyotype (58XY; clone 24 and 27; Fig. 1e), telomerase activity was high (Supplementary Fig. 1d), exponential proliferation occurred for ≥77 days (Supplementary Fig. 1e) and the cells remained undifferentiated at passage 30. The growth rates of NMR-iPSCs and parental fibroblasts were similar (Supplementary Fig. 1f). Quantitative real-time polymerase chain reaction (qRT–PCR) using the primers specific for OSKM transgenes showed decreases in the expression of all four retroviruses (Supplementary Fig. 1g). AP activity, RNA-sequencing (RNA-seq), reverse transcription polymerase chain reaction (RT–PCR) analysis and immunocytochemistry revealed upregulation of several pluripotency markers and downregulation of fibroblast markers (Fig. 1d,f; Supplementary Fig. 1h,i). Principal component analysis of global gene expression patterns of RNA-seq data showed that four NMR-iPSC clones clustered together and were distinct from parental fibroblasts (Supplementary Fig. 1j). We next determined the developmental potential of NMR-iPSCs by embryoid body (EB) formation. Immunocytochemistry and RT–PCR analyses revealed that NMR-iPSCs differentiated into cells of the three germ layers (Fig. 1g and Supplementary Fig. 1k). To clarify whether NMR-iPSCs acquire tumorigenicity and form teratomas *in vivo*, we transplanted these cells into the testes of NOD/SCID mice. In contrast to control Ms- and human-iPSCs, NMR-iPSCs did not form tumours (Fig. 1h,i, Supplementary Fig. 2a,b). GFP-positive NMR-iPSCs engrafted in the testis without forming tumours (Supplementary Fig. 2c). These results suggest that reprogramming to a pluripotent state can be induced in cancer-resistant NMRs without inducing tumorigenicity.

### The role of ARF and ERAS in tumour resistance of NMR-iPSCs

**Figure 2:**
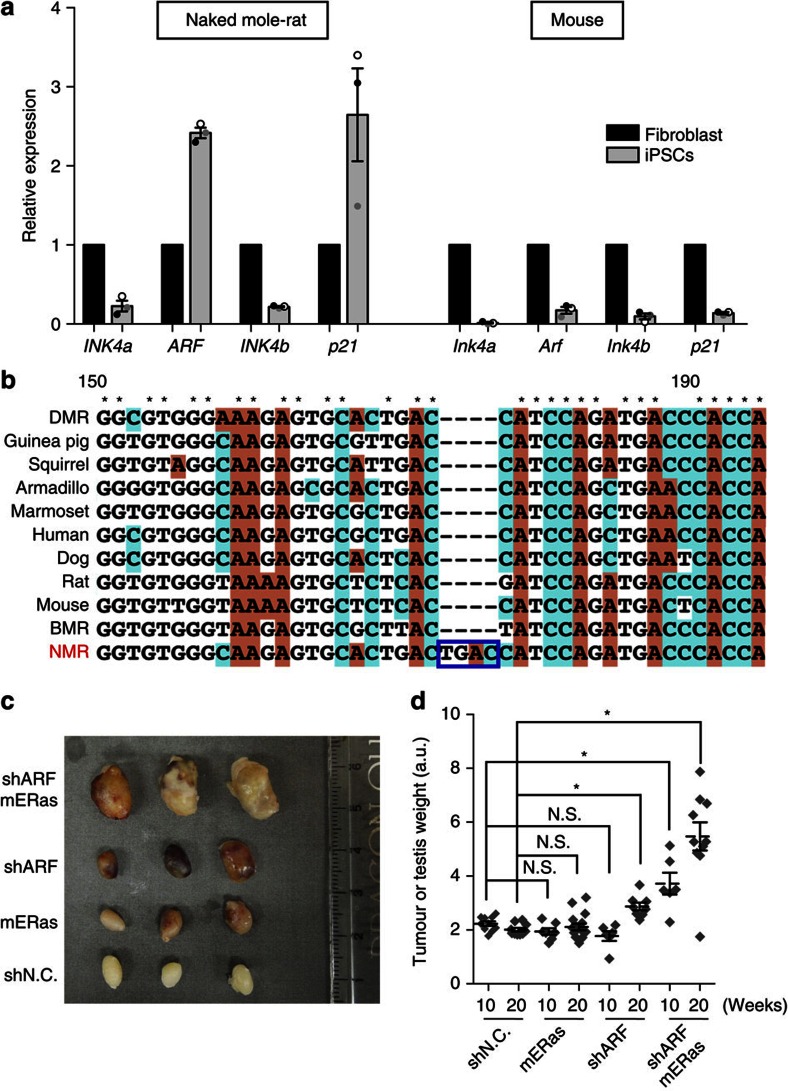
Activation of *ARF* and loss-of-function mutation in *ERAS* regulate tumour resistance of NMR-iPSCs. (**a**) The qRT–PCR analysis of the expression of *INK4a, ARF, INK4b* and *p21* in NMR- and Ms-iPSCs (*n*=3 clones). The data are represented as mean±s.e.m. (**b**) Alignment of the coding sequences of *ERAS* of 11 species. DMR, damaraland mole-rat; BMR, blind mole-rat. (**c**,**d**) Teratoma formation. shN.C.-, shARF-, mERas- or shARF/mERas-expressing NMR-iPSCs were transplanted into the testes of NOD/SCID mice. Representative tumours 10 weeks after transplantation (**c**). Tumour weights (**d**). *n*=8 (shN.C., 10 weeks; shN.C., 20 weeks; mERas, 10 weeks; shARF, 10 weeks; shARF/mERas, 10 weeks), *n*=10 (shARF/mERas, 20 weeks) and *n*=16 (mERas, 20 weeks; shARF, 20 weeks). *n*: number of transplanted testes. The data are represented as mean±s.e.m. *Y* axis: weights in 6+log_2_ arbitrary units. *P<0.05; NS, not significant, Kruskal–Wallis followed by Dunn's test.

To identify genes contributing to tumour resistance in NMR-iPSCs, we used RNA-seq and qRT–PCR and analysed the expression levels of genes associated with NMR tumour resistance and ES/iPSC tumorigenicity^16^,^18^,^19^,^20^,^21^,^22^,^23^,^24^. The expression levels of both *hyaluronan synthase 2* (*HAS2*) and *pALT*^*INK4a/b*^, which negatively contribute to tumorigenicity in NMR-fibroblasts^18^,^19^, were quite lower in NMR-iPSCs (Fig. 1f, Supplementary Fig. 3a,b). In contrast to mice, we found that the expression of the *ARF* and its downstream gene *p21* were higher in NMR-iPSCs relative to expressions of the same genes in NMR fibroblasts, whereas expression of *INK4a* and *INK4b* were suppressed (Fig. 2a, Supplementary Fig. 3c). The *INK4/ARF* locus is known to frequently be genetically or epigenetically inactivated in human cancers, and also to be strongly suppressed in human- and Ms-iPSCs^13^,^14^,^15^. Ms-iPSCs derived from transgenic mice carrying an extra copy of the *Ink4/Arf* locus exhibits reduced tumorigenicity, although extra copy of *Ink4a/Arf* are not active in non-stressed undifferentiated cells^24^.

Next, we determined that NMR-iPSCs do not express oncogenic *ERAS*, which positively regulates the tumorigenicity of Ms-ESCs through activation of the phosphoinositide 3-kinase/AKT pathway^16^ (Supplementary Fig. 3d). NMR-*ERAS* harbours a mutation that introduces a premature stop codon that removes the carboxy (C)-terminal CAAX motif required for its transforming activity (Fig. 2b and Supplementary Fig. 3e). NMR-ERAS did not induce the transformation of NIH-3T3 cells (Supplementary Fig. 4a–f).

To evaluate the roles of ARF activation and ERAS disruption in tumour resistance in NMR-iPSCs, we introduced short hairpin RNAs targeting *ARF* (shARF) and/or Ms-*ERas* (mERas) into NMR-iPSCs (shARF-, mERas- or shARF/mERas-NMR-iPSCs). No changes were observed in the morphology of shARF-NMR-iPSCs, while mERas- and shARF/mERas-NMR-iPSCs formed flattened colonies (Supplementary Fig. 5a,b). We confirmed p21 suppression and induction of AKT phosphorylation by introducing shARF and mERas, respectively (Supplementary Fig. 5c,d). The phosphorylation level of AKT in NMR-iPSCs on ectopic expression of *mERas* was similar to that in Ms-iPSCs (20D17) and Ms-ESCs (EGR-G101), indicating that the AKT phosphorylation level in mERas-NMR-iPSCs was not supra-physiological. Moreover, the AKT phosphorylation levels in NMR-iPSCs and *ERas* knocked-out Ms-ESCs were low compared with that in control Ms-ESCs (Supplementary Fig. 5d). A heat map showing hierarchical clustering analysis of the expression patterns of selected markers of undifferentiated and differentiated states from RNA-seq data suggested that additional reprogramming did not occur following the introduction of shARF- and/or mERas (Supplementary Fig. 5e–g).

We then tested anchorage-independent growth in soft agar, which is a conventional assay used to detect tumorigenicity *in vitro* (Supplementary Fig. 5h,i). Control or mERas-NMR-iPSCs remained as single cells or formed a few small colonies, whereas shARF-NMR-iPSCs formed significantly higher numbers of small colonies. shARF/mERas-NMR-iPSCs formed colonies larger than those of shARF-NMR-iPSCs, indicating that anchorage-independent growth potential depended on the inactivation of ARF and that mERas enhanced cell proliferation. These were transplanted into NOD/SCID mice testes to evaluate *in vivo* tumorigenic potential. The shARF-NMR-iPSCs were more tumorigenic than mERas-NMR-iPSCs, and shARF/mERas-NMR-iPSCs formed large tumours (Fig. 2c,d, Supplementary Fig. 6a,b). Tumours derived from shARF/mERas-NMR-iPSCs were teratomas that differentiated into cells of the three germ layers (Supplementary Fig. 6c,d). Thus, *ARF* activation and disruption of *ERAS* regulates the tumour resistance of NMR-iPSCs.

### Forced expression of Arf reduces tumorigenicity of Ms-iPSCs

**Figure 3:**
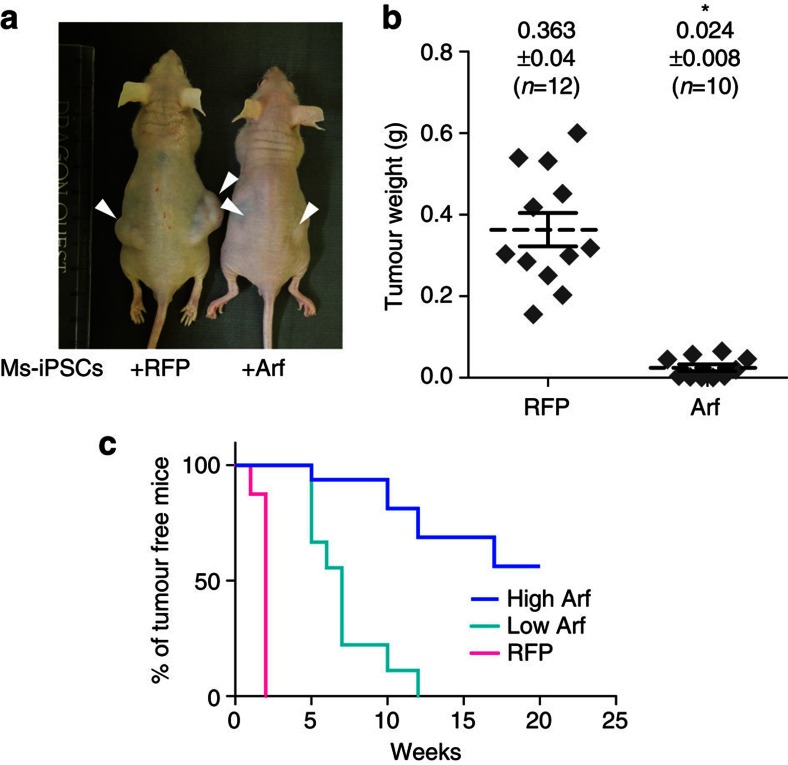
Ectopic expression of *Arf* significantly attenuates the tumourigenicity of Ms-iPSCs. (**a**) Nude mice transplanted subcutaneously with Ms-iPSCs expressing red fluorescent protein (RFP; left) or Arf (right) 5 weeks after transplantation. Arrowhead: transplantation site. (**b**) Comparison of tumour weights 3 weeks after transplantation. Unpaired *t*-test; *n*=12 transplanted sites. The data are represented as mean±s.e.m. (**c**) Kaplan–Meier curve of tumour-free mice transplanted with Low-Arf-group Ms-iPSC clones (clone 5 and 6, *n*=8 mice, turquoise line), High-Arf-group Ms-iPSC clones (clone 2, 3 and 4, *n*=16 mice, blue line) or control Ms-iPSCs expressing RFP (*n*=8 mice, magenta line), respectively.

Next, we stably expressed Arf in Ms-iPSCs derived from a *Nanog*-EGFP reporter mouse^25^ (Arf-Ms-iPSC) to study whether this reduced the tumorigenicity of Ms-iPSCs, and found that it had little effect on undifferentiated marker expression (Supplementary Fig. 7a,b). To evaluate tumorigenicity, we first classified *Arf* transgenic cell clones into High-Arf (No. 2, 3, 4) and Low-Arf (No. 5, 6) groups, as defined by *Arf* expression levels without drug selection (Supplementary Fig. 7c). Although tumour formation was markedly reduced in both the Low- and High-Arf groups (Fig. 3a,b), the High-Arf group acquired significantly higher tumour resistance than the Low-Arf group; 56.25% of High-Arf group mice survived ≥14 weeks without detectable tumours and 7 of 42 injection sites (16.67%) of High-Arf group mice developed tumours (Fig. 3c, Supplementary Fig. 7d). Haematoxylin–eosin staining showed that these tumours were teratomas (Supplementary Fig. 7e). *Arf* transgene expression was strongly suppressed in tumours compared with undifferentiated Arf-Ms-iPSCs, possibly due to gene silencing (Supplementary Fig. 7f). EB formation showed that the High-Arf group had differentiation potential into derivatives of all three germ layers *in vitro* (Supplementary Fig. 8a,b).

In mice and humans, expression of both *INK4a* and *ARF* is initially upregulated, and then strongly suppressed in the later stages of reprogramming^14^. To determine why NMR-iPSCs retain relatively high *ARF* expression and exhibit tumour resistance, we analysed the kinetics of *INK4a* and *ARF* expression during reprogramming. *INK4a* and *ARF* were derepressed during early reprogramming. In late reprogramming, *INK4a* was suppressed (as it is in both mice and humans) and *ARF* expression was retained in NMR-iPSCs (Supplementary Fig. 9a). Next, we analysed whether *ARF* was upregulated in response to c-MYC activation^26^ in NMR-iPSCs, and found that the total c-MYC level was lower than it was in NMR-fibroblasts, and that the expression of genes regulated by c-MYC was similar to expression of those genes in parental fibroblasts (Supplementary Fig. 9b,c).

### ARF-suppression blocks iPSC generation by NMR-specific ASIS

**Figure 4:**
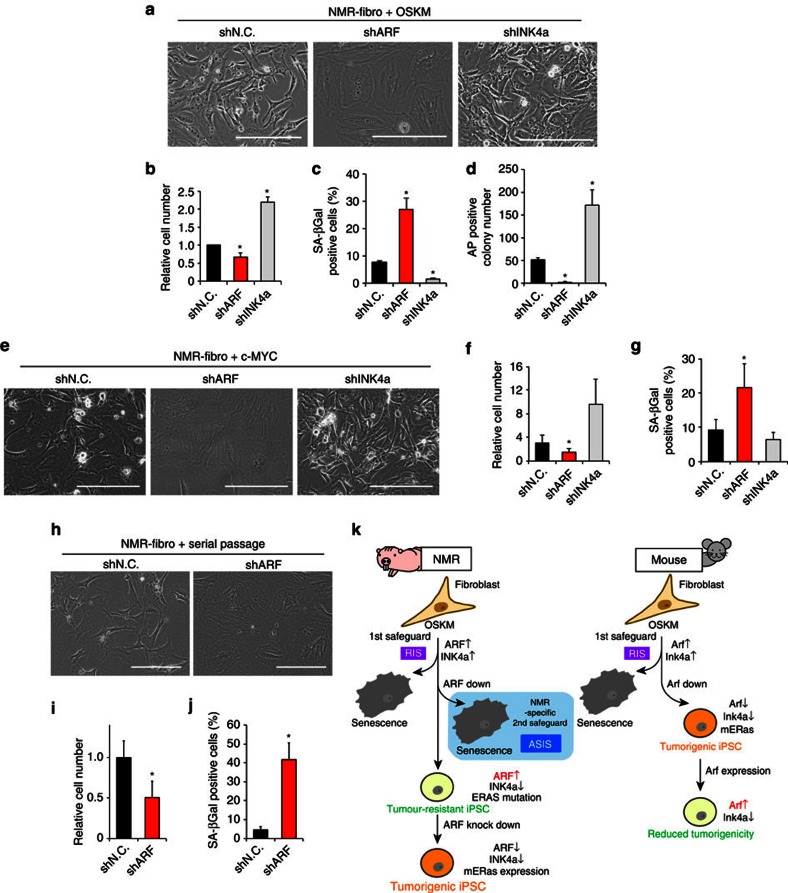
Suppression of ARF induces NMR-specific cellular senescence as a safeguard against reprogramming and oncogenic transformation. (**a**–**d**) Co-transduction of NMR-fibroblasts with shARF or shINK4a with the OSKM; cell morphology (**a**), cell growth (**b**), SA-βGal-positive cells (%) (**c**), AP-positive colonies (**d**). (**e**–**g**) Transduction of NMR-fibroblasts overexpressing c-Myc with shARF or shINK4a; cell morphology (**e**), cell growth (**f**), SA-βGal-positive cells (%) (**g**). (**h**–**j**) Transduction of shARF of serially-passaged NMR-fibroblasts; cell morphology (**h**), cell growth (**i**), SA-βGal-positive cells (%) (**j**). (**k**) Role of ARF and ERAS in reprogramming without acquisition of tumorigenicity in NMR-iPSCs. RIS, reprogramming-induced senescence. ASIS, ARF suppression-induced senescence. Scale bar, 200 μm. Results are presented as mean±s.d. for three biological replicates. *P<0.05 between the indicated groups (one-way analysis of variance (ANOVA) for **b**–**d**,**f**, and **g**; *t*-test for **i** and **j**.

To evaluate the effects of ARF downregulation on NMR-fibroblasts, we performed *ARF* knockdown during reprogramming; this has been shown to enhance reprogramming efficiency in mice^13^,^14^,^15^. In NMR-fibroblasts, ARF suppression induced a phenotype similar to that of senescent cells, including enlarged cytoplasm and activation of senescence-associated β-galactosidase (SA-βGal) activity, resulting in the inhibition of reprogramming. In contrast, *INK4a* knockdown enhanced cellular growth and reprogramming efficiency exhibited by Ms and human cells (Fig. 4a–d, Supplementary Fig. 9d,e). Furthermore, *ARF* knockdown in stressed NMR-fibroblasts, in which ARF had been derepressed by either *c-MYC* oncogene overexpression or serial passage, induced cellular senescence (Fig. 4e–j, Supplementary Fig. 9f–j). We termed this phenomenon ‘ARF suppression-induced senescence' (ASIS). To gain mechanistic insight into ASIS induction, we examined the activation status of RB, AKT, MAPK and several cell cycle inhibitors, which are reported to regulate cellular senescence (Supplementary Fig. 10a,b). A previous study has shown that both RB hypo-phosphorylation by Ink4a and AKT phosphorylation by mitogenic signalling are both required for the induction of cellular senescence in Ms-fibroblasts^27^. Furthermore, INK4a is reported to be essential for the maintenance of cellular senescence^28^,^29^. However, we found that in the NMR fibroblasts undergoing ASIS, RB hypo-phosphorylation was induced without INK4a, p21 and p27 upregulation, whereas AKT was phosphorylated along with ERK activation. The absence of the involvement of cell cycle inhibitors (such as INK4a, p21 and p27) is the interesting feature of ASIS, and other genes may regulate ASIS as NMR-specific safeguard.

## Discussion

Cells suffer from stressors such as reprogramming, oncogene activation, and replication stress are known to derepress INK4a and ARF expression^30^. Stressed cells become senescent; this is the first safeguard against oncogenic transformation^30^,^31^. Given the results of the present study, we suggest that NMR-specific ASIS may act as a second safeguard, inducing cellular senescence when ARF is suppressed in cells, in which ARF has been derepressed by exposure to stressors (Fig. 4k). An ARF-activated cell population may thus be selected during the generation of NMR-iPSCs. Moreover, NMRs encode truncated forms of *INK4a* and *ARF*^20^,^32^, suggesting that unique cancer-resistance mechanisms mediated by INK4a and ARF evolved in NMRs.

We conclude that the tumour resistance in NMR-iPSCs is based on NMR-specific ARF regulation and disruption of ERAS. Further research into the detailed mechanisms underlying ASIS in NMRs may contribute to the generation of non-tumorigenic human-iPSCs enabling safer cell-based therapeutics and shed new light on cancer resistance in the naked mole-rat.

## Methods

### Animals

The Ethics Committees of Hokkaido University (Approval no. 14-0065) and Keio University (approval no. 12,024) approved all the procedures, which were in accordance with the Guide for the Care and Use of Laboratory Animals (United States National Institutes of Health, Bethesda, MD). The NMR colonies are maintained at Hokkaido University. C57BL/6 mice and BALB/c nude mice were purchased from CLEA Japan, Inc. The NOD/SCID mice were purchased from Charles River. The cells and tissues were obtained from at least two animals.

### Statistical analysis

The data are presented as the mean±s.e.m. or mean±s.d. The data were analysed using one-way analysis of variance followed by the analysis of variance test or the Kruskal–Wallis nonparametric test followed by Dunn's test for multiple comparisons or the unpaired *t*-test for two groups. Graphpad Prism was used for statistical analysis.

### Cell culture and retroviral infection

NMR- or Ms-skin fibroblasts were isolated from 1- to 2-year-old adult male NMRs or 6-week-old adult male C57BL/6 mice. The skin including the epidermis was washed with phosphate-buffered saline (PBS) containing 1% penicillin/streptomycin (Wako) and amphotericin B (Wako) and then treated with 0.25% trypsin/EDTA (Wako) and 5 mg ml^−1^ collagenase (GIBCO) at 32 °C for 30 min. The reaction was stopped by adding 15% fetal bovine serum (FBS) medium (contents described below). The cell clumps and minced tissues were collected by centrifugation (180*g*, 5 min), resuspended in fresh medium, plated on gelatin-coated 10-cm cell culture dishes and cultured at 32 °C (NMR-fibroblasts) or 37 °C (Ms-fibroblasts) in a humidified atmosphere containing 5% CO_2_. The cells were cultured in 15% FBS medium composed of Dulbecco's Modified Eagle's Medium (DMEM, Sigma Aldrich) supplemented with 15% FBS (JRH or BioWest), 1% penicillin/streptomycin, 2 mM L-glutamine (Nakalai Tesque) and 0.1 mM non-essential amino acids (NEAA, Nakalai Tesque).

We used a lentiviral vector to transduce NMR-fibroblasts (passage 2) with the Ms retroviral receptor Slc7a1 (ref. 33). The cells were seeded at 1.5 × 10^5^ cells per 10-cm dish 1 day before transduction; 3 days later, the NMR-fibroblasts (passage 3) were transduced with retroviral vectors that express Ms OSKM, as previously described^7^. In brief, pMXs-based retroviral vectors were introduced into Plat-E cells with Fugene 6 transfection reagent (Roche) according to the manufacturer's instructions. Twelve hours after transduction, the medium was replaced. Another 24 h later, the conditioned medium containing virus particles derived from these Plat-E cultures was used for viral transduction. A second retroviral infection was performed 4 days after the first because of the low infection efficiency of NMR-fibroblasts. Nine days after the first transduction, fibroblasts were plated at 1.5 × 10^5^ cells per 10-cm dish on a mitomycin C-treated SNL-STO (MSTO) feeder layer. The medium was replaced with standard human ES medium containing DMEM/F12 (Sigma Aldrich) supplemented with 20% knockout serum replacement (Life Technologies), 1% penicillin/streptomycin, 2 mM L-glutamine, 0.1 mM NEAA, 0.1 mM β-mercaptoethanol (2-ME, Sigma Aldrich) and 4 ng ml^−1^ fibroblast growth factor 2 (PeproTech). The medium was changed every day, and we isolated iPSC-like colonies 30 days after reseeding on feeder layers.

NMR-iPSCs were maintained on MSTO feeder layers on gelatin-coated six-well plates, passaged once each week and reseeded at 2.0 × 10^5^ cells per well. Ms-iPSCs (20D17, 38C2, N3E6; refs 25, 34) were cultured in standard Ms-ES medium containing DMEM supplemented with 15% FBS, 1% penicillin/streptomycin, 2 mM L-glutamine and 0.1 mM NEAA, 0.1 mM 2-ME and human recombinant leukaemia inhibitory factor (LIF, Nacalai Tesque) or conditioned medium containing LIF produced by Plat-E cell cultures transfected with a vector encoding human LIF (pCAGGS-hLIF)^35^.

### Karyotyping

Chromosomal G-band analyses were performed at Nihon Gene Research Laboratories. The karyotypes of NMR-iPSC clones 24 and 27 were normal and that of clone 12 was tetraploid.

### Molecular cloning of NMR-*ERAS* coding sequence

PCR was performed using primers, shown in Supplementary Table 1, designed according to the genomic sequences of NMR-*ERAS* deposited in the database of the Beijing Genomics Institute (BGI)^20^. DNA fragments were inserted into the pENTR/D-TOPO entry vector and sequenced using the Sanger method. The NMR-*ERAS* sequence (NCBI Reference Sequence: XM_004921208) previously deposited has one base deletion to adjust frame by automated computational analysis using gene prediction method (Gnomon).

### RT–PCR analysis

To remove the feeder-layer MSTO cells, dissociated iPSCs were allowed to adhere for 20 min to a gelatin-coated dish. The supernatant containing the iPSCs was centrifuged. Total RNA of iPSCs and fibroblasts were purified using Trizol reagent (Invitrogen) and treated with a Turbo DNA-free kit (Ambion) to remove contaminating genomic DNA. Total RNA (500 ng) was reverse-transcribed using ReverTraAce (Toyobo) according to the manufacturer's instructions. PCR was performed using ExTaq-HS polymerase (Takara). The primer sequences are listed in Supplementary Table 1.

### qRT–PCR analysis

Ms and NMR cells were collected and washed with ice-cold PBS. The cells were centrifuged, and total RNA was extracted from the pellet using the RNeasy Plus Mini Kit (Qiagen). We used gDNA Eliminator spin columns to remove genomic DNA. The complementary DNA (cDNA) was synthesized using the ReverTra Ace qRT–PCR RT Master Mix (Toyobo) with 400 ng of total RNA. The qRT–PCR was performed in triplicate with SYBR Premix Ex Taq II (Takara) or Fast SYBR Green Master Mix (Invitrogen) using a ViiA 7 or StepOne plus Real-Time PCR System (Applied Biosystems). Primer sequences are listed in Supplementary Table 1.

### AP activity and immunocytochemistry

AP activity was measured using an Alkaline Phosphatase Detection Kit (System Biosciences) according to the manufacturer's instructions. For immunocytochemistry, NMR-iPSCs and differentiated cells were fixed with 4% paraformaldehyde in PBS for 5 min at room temperature, washed with PBS and then treated with 0.3% Triton X-100 in Tris-NaCl-blocking buffer (PerkinElmer) for 60 min at room temperature. The cells were incubated with primary antibodies against Oct4 (Millipore; 7F9.2; 1:200) and E-cadherin (BD Pharmingen; clone 36; 1:500) for 12 h at 4 °C. After washes with PBS, the cells were incubated with secondary antibody Alexa Fluor 555 anti-Ms IgG (Cell Signaling Technology (CST); A21424; 1:1,000) and Alexa Fluor 555 anti-rabbit IgG (CST; A21429; 1:1,000), and nuclei were counterstained with 1 μg ml^−1^ Hoechst 33,258 (Sigma Aldrich) for 60 min at room temperature. After washes with PBS, the images were captured.

### *In vitro* differentiation

EB formation was analysed by dissociating the NMR-iPSCs with 0.1% trypsin-EDTA and plating them on an ultra-low attachment coated dish (Corning) in human ES medium without basic fibroblast growth factor. After 14 days, EBs were transferred to gelatin-coated adhesion dishes and cultured for 7 days. Culture medium was replaced every second day. On day 21, the cells were fixed with 4% paraformaldehyde and analysed using the antibodies against the proteins as follows: GFAP (Dako; Z0334; 1:4,000), NESTIN (non-commercial^36^,^37^; 1:1,000), αSMA (Sigma Aldrich; 1A4; 1:1,000), DESMIN (Thermo Scientific; RB-9014; 1:1,000), FOXA2 (ABNOVA; 7E6; 1:1,000) and VIMENTIN (abcam; EPR3776; 1:1,000).

Ms-iPSCs were dissociated with 0.25% trypsin-EDTA and plated onto an ultra-low attachment coated dish in 10 ml of αMEM (Gibco) supplemented with 10% FBS and 0.1 mM 2-ME (EB medium), cultured for 6 days, transferred to a poly-L-ornithine (Sigma Aldrich)/fibronectin (Sigma Aldrich)-coated adhesion dish and cultured for 24 h. To assess direct neural differentiation, Ms-iPSCs were dissociated and then plated onto an ultra-low-coated attachment dish in 10 ml of EB medium. On day 2, 10^−8^ M retinoic acid (Sigma Aldrich) was added to the culture medium, and after 4 days, EBs were transferred to a poly-L-ornithine/fibronectin-coated adhesion dish and cultured in medium containing DMEM/F-12 (Gibco), 0.6% glucose, 2 mM L-glutamine, 3 mM sodium bicarbonate, 5 mM HEPES, 25 μg ml^−1^ insulin (Sigma Aldrich), 100 μg ml^−1^ transferrin (Nakalai Tesque), 20 nM progesterone (Sigma Aldrich), 30 ng sodium selenate (Sigma Aldrich) and 60 nM putrescine (Sigma Aldrich) and cultured for 24 h as previously described^38^. For immunocytochemical analyses, the cells were fixed with 4% paraformaldehyde and analysed using the antibodies against the proteins as follows: αSMA (Sigma Aldrich; 1A4; 1:1,000), albumin (Sigma Aldrich; A0433; 1:1,000), vimentin (Abcam; EPR3776; 1:1,000), Nestin (BD Pharmingen; BD-556309; 1:250), Tubb3 (Sigma Aldrich; T8660; 1:1,000).

### Telomerase activity

Telomerase activity was detected using the TRAPeze Telomerase Detection Kit (Chemicon), according to the manufacturer's instructions. Telomere repeat additions were performed at 30 °C for 120 min. The samples were electrophoretically separated through 20% polyacrylamide gels in 0.5 × TBE. The gel was stained with SYBR Gold (Invitrogen; 1:10,000).

### RNA-seq

Total RNA was extracted from NMR-fibroblasts and NMR-iPSCs using TRIzol reagent (Invitrogen) followed by Qiagen RNeasy column purification. The quality and quantity of the RNA preparations were assessed using a 2100 Bioanalyzer with an RNA 6000 Nano LabChip Kit (Agilent Technologies). Poly(A)+ RNA was selected and converted to a library of cDNA fragments (200–250 bp) with adaptors attached to both ends for sequencing using a TruSeq RNA Sample Prep Kit v2 (Illumina), as per the manufacturer's instructions. Libraries were quantified using a Bioanalyzer DNA High Sensitivity Kit (Agilent Technologies) and Kapa Library Quantification Kit (Kapa Biosystems) using an Applied Biosystems StepOne Real-Time PCR System, according to the manufacturer's instructions. The libraries were then loaded into a flow cell for cluster generation using the TruSeq Rapid SR Cluster Kit (Illumina) and sequenced using an Illumina HiSeq2500 to obtain 100-nucleotide sequences (single-end).

Base-calling and Chastity filtering were performed using real-time analysis software version 1.18.61. Illumina fastq files generated using real-time analysis were trimmed with cutadapt (http://code.google.com/p/cutadapt/) to remove the Illumina Truseq adapter sequence, and sequences with ≥50 nucleotides were selected. The fastq sequences of NMR-iPS and Ms fibroblasts were separated using xenome (http://www.genomics.csse.unimelb.edu.au/product-xenome.php) from the trimmed fastq files. The NMR reference sequence files and annotation general feature format file were downloaded from BGI ftp site (ftp://ftp.genomics.org.cn/pub/Heterocephalus_glaber/).

We used bowtie build v.0.12.9 to build Burrows–Wheeler transform indexes for the reference genomic sequence, which were combined with data from genomic and mitochondrial sequence files. The trimmed NMR fastq files were aligned to the reference genomic sequence using TopHat v.2.0.12 (http://tophat.cbcb.umd.edu/) with SAMtools v.0.1.18 and non-default parameters as follows: --num-threads 6 --max-multihits 1 --transcriptome-index=‘BGI naked mole-rat cdna sequence'.

Transcript abundances were calculated and fragments per kilobase of transcript per million mapped reads-normalized to the upper quartile using Cufflinks v.2.0.12 (http://cufflinks.cbcb.umd.edu/). The differential expression of transcripts by fibroblasts and iPSCs was estimated using Cuffdiff. Heat maps of the differentially expressed genes were generated using the heatmap.3 function in the plots package of R. We selected 31 pluripotency-related genes^7^,^8^,^39^ and 15 fibroblast-marker genes shown in Fig. 1f and Supplementary Fig. 5e–g. In Supplementary Fig. 9c, c-Myc-target genes were selected, as previously described^40^.

### Cell proliferation

NMR-iPSCs and -fibroblasts were passaged every 7 days and replated at 2 × 10^5^ per six-well plate and 3 × 10^5^ per 10-cm dish, respectively. Reseeded cells were counted using a Coulter Counter (Beckman Coulter). The population doubling time, including cell cycle arrest and cell death, was calculated from the slopes of growth curve.

### Lentivirus preparation

We used the lentiviral vectors pCSII-EF-NMR-ERAS-TK-hyg, pCSII-EF-mERas-TK-hyg, pCSII-EF-HRasV12-TK-hyg, pCSII-EF-NMR-c-MYC-TK-hyg or pCSII-EF-EGFP-TK-hyg for ectopic expression and H1 promoter-driven vectors previously described^41^ for shRNA expression. Three knockdown vectors expressing shRNA of NMR-ARF were generated and validated as previously shown^32^. The target sequences of shRNAs are shown in Supplementary Table 2. Each plasmid and packaging vectors (pCMV-VSV-G-RSV-Rev and pCAG-HIVgp)^41^ were used to transfect 293T cells with polyethylenimine MAX transfection reagent (CosmoBio), according to the manufacturer's instructions. The conditioned medium containing virus particles was concentrated and used for viral transduction.

### CRISPR/Cas9-mediated gene disruption

A 20-nt guide RNA (gRNA) sequence (5′-CATGGTCTTTCACGAAGCAT-3′) was designed to target double-strand breaks at protein coding region of *ERas* and cloned into the Cas9 and sgRNA expression vector SpCas9-2A-Puro (Addgene, PX459 #62988; ref. 42). The plasmid was transfected into the Ms-ESC lines (EGR-G101; ref. 43) using CUY21EDIT2 (BEX) according to the manufacturer's protocol. The transfected cells were enriched by 2 days of puromycin selection starting 24 h after transfection and colonies were picked up. DNA was extracted from each colony and coding sequence for *ERas* was amplified and sequenced using the Sanger method.

### Stable ectopic expression of Arf in Ms-iPSCs

Ms-iPSCs expressing Arf were generated by electroporating cells with the pCAG-Arf-IRES-hyg vector and selecting hygromycin-resistant cells for 7 days, and then Arf-Ms-iPSC colonies were picked up. The differentiation potential of EBs derived from the Arf-Ms-iPSCs was evaluated by qPCR for differntiation marker genes^44^.

### Teratoma formation

iPSCs were suspended (1 × 10^7^ cells per 200 μl) in medium. NOD/SCID mice were anaesthetised using isoflurane. We injected 20 μl of the cell suspension (1 × 10^6^ cells) into each testis. Ten, 20 or 28 weeks after transplantation, tumours and testes were dissected, fixed overnight in PBS containing 4% paraformaldehyde and embedded in paraffin. The sections were stained with haematoxylin and eosin or subjected to immunohistochemical analysis to detect the expression of EGFP and differentiation markers. The sections were treated with 0.1 M citrate buffer at 105 °C for 20 min and incubated at 4 °C overnight with antibodies against the following proteins: GFP (MBL; 598; 1:500), αSMA (Sigma Aldrich; 1A4; 1:500), VIMENTIN (Abcam; EPR3776; 1:1,000), NESTIN (non-commercial antibody^36^,^37^; 1:1,000) and GFAP (Dako; Z0334; 1:1,000). After washes with PBS, the slides were incubated with secondary antibodies conjugated to horseradish peroxidase (HRP) anti-Ms IgG (CST; 7,076; 1:1,000) and anti-rabbit IgG (CST; 7,074; 1:1,000) for 1 h at room temperature, washed with PBS and antigen–antibody complexes were detected using DAB (Vector Laboratories).

The tumorigenicity of Ms-iPSCs was assessed by injecting subcutaneously 5 × 10^5^ cells into nude mice. The mice were monitored once each week for teratoma formation and general health and killed 3 or 5 weeks later. To determine the tumour-free survival rate, we monitored the mice once each week. Scoring criterion: mice with visible tumours were designated ‘tumour-positive'.

### Growth in soft agar

To test for anchorage-independent growth, NMR-iPSCs were suspended to 3 × 10^3^ cells per 3 ml of 0.35% agar in growth medium and poured over a solidified layer of 0.65% agar medium in a six-well plate. Three weeks later, the cell colonies were stained using toluidine blue and enumerated.

### SA-βGal activity

SA-βGal activity was measured using the Senescence Detection Kit (BioVision). Cells were stained for 48 h at 37 °C according to the manufacturer's instructions. The cells were washed with PBS and stained with Hoechst 33,258 (diluted 1:1,000 with PBS) for 30 min at room temperature in the dark. The cells were washed in PBS and analysed by microscopy. The cell populations in at least three random fields (≥500 cells) were analysed for perinuclear blue staining indicative of SA-βGal activity, and the nuclei were visualized using Hoechst staining.

### Western blotting

The cells were washed with PBS, lysed in cell-lysis buffer (62.5 mM Tris-HCl, pH 6.8; 2% SDS and 5% sucrose) and boiled for 5 min. The protein concentrations were measured using a Pierce BCA Protein Assay Kit (Thermo Scientific). The samples were subjected to SDS–PAGE (polyacrylamide gel electrophoresis), and the proteins were transferred to a polyvinylidene fluoride membrane using a Trans-Blot Turbo Transfer System (Bio-Rad). The membranes were probed with antibodies against NMR-INK4a (non-commercial^32^; 1:1,000), NMR-ARF (non-commercial^32^; 1:10,000), AKT (CST; 9,272; 1:1,000), pAKT (CST; 4,060; 1:1,000), p53 (Sigma Aldrich; AV02055; 1:1,000), p21 (BD Biosciences; 555,430; 1:1,000), p27 (CST; 3,698; 1:1,000), RB (CST; 9,309; 1:1,000), pRB (CST; 8,516; 1:1,000), ERK (CST; 9,102; 1:1,000), pERK (CST; 4,370; 1:1,000), p38 (CST; 9,212; 1:1,000), p-p38(CST; 4,511; 1:1,000) and beta-actin (Sigma Aldrich; AC-15; 1:100,000). The membranes were incubated with HRP-conjugated anti-rabbit (CST; 7,074; 1:1,000) or anti-Ms (CST; 7,076; 1:1,000) IgG secondary antibodies and visualized using enhanced chemiluminescence (ECL; GE Healthcare). All uncropped western blots can be found in Supplementary Fig. 11.

## Additional information

**Accession codes:** RNA-seq data have been deposited in the DNA Data Bank of Japan (DDBJ) database under accession code: DRA003980. The sequence of NMR-*ERAS* was deposited in the GenBank database under accession code: LC074725.

**How to cite this article:** Miyawaki, S. *et al*. Tumour resistance in induced pluripotent stem cells derived from naked mole-rats. *Nat. Commun.* 7:11471 doi: 10.1038/ncomms11471 (2016).

## Supplementary Material

Supplementary Information Supplementary Figures 1-11 and Supplementary Tables 1-2
