# Supplementary material for: Tumour resistance in induced pluripotent stem cells derived from naked mole-rats
Source: Nat Commun. 2016 May 10;7:11471. doi: 10.1038/ncomms11471 (PMC4866046; doi:10.1038/ncomms11471)
Supplement: Supplementary Information — Supplementary Figures 1-11 and Supplementary Tables 1-2 [file ncomms11471-s1.pdf]

Supplementary Figure 1

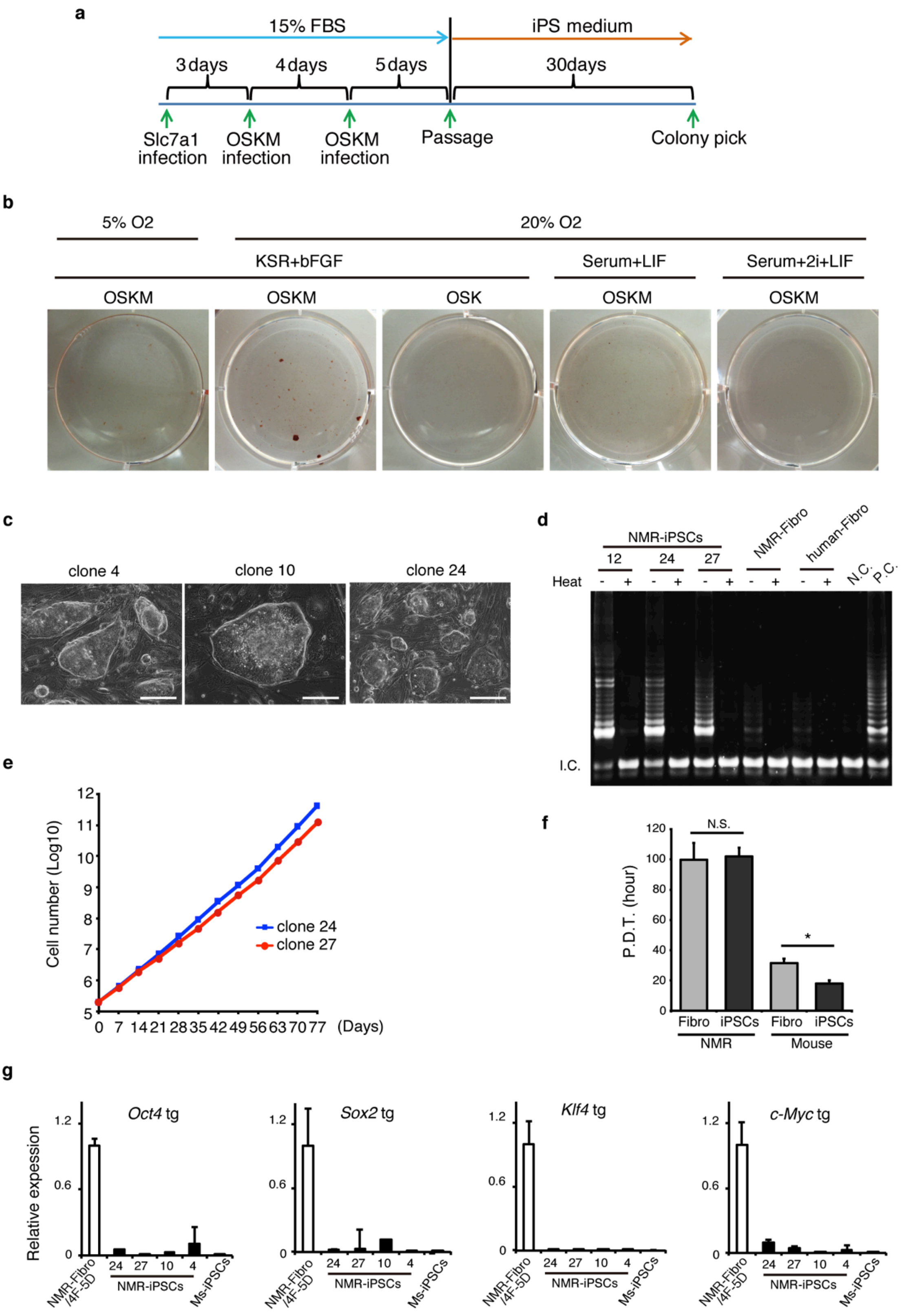

Supplementary Figure 1

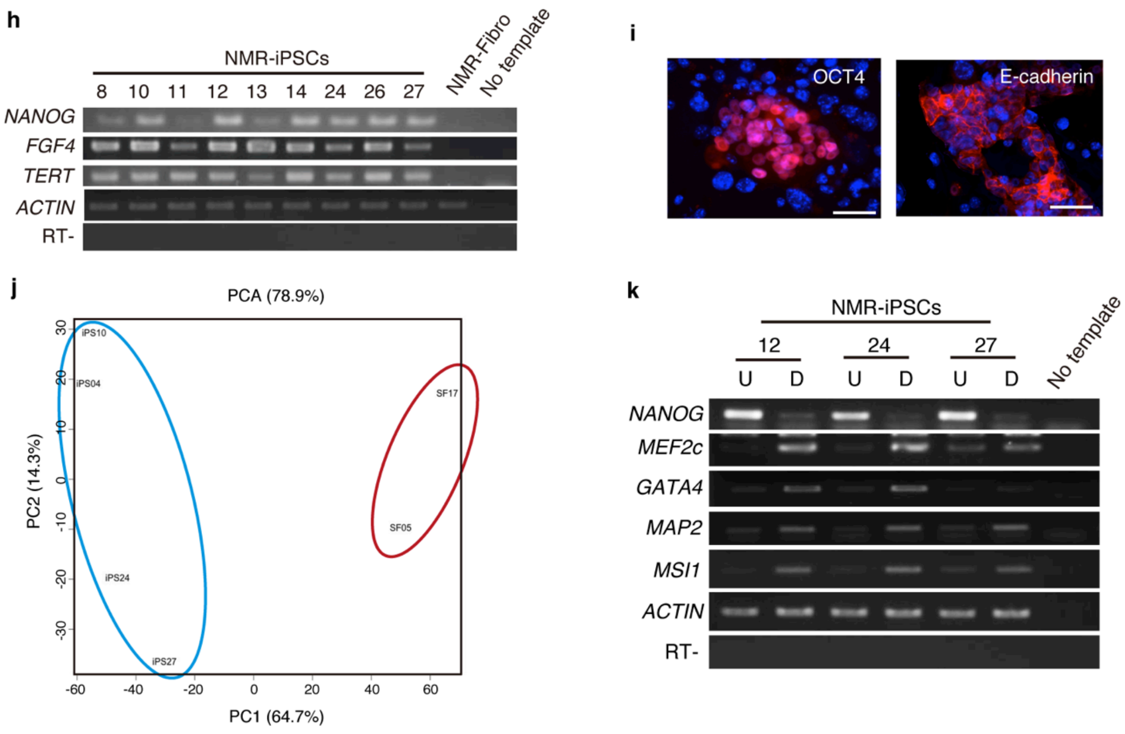

Supplementary Figure 1 | Generation and characterization of NMR-iPSCs.

a, iPSC generation from NMR-fibroblasts. **b**, Culture conditions. AP activity 37 days after introduction of OSKM. **c**, Morphology of NMR-iPSCs (clones 4, 10 and 24). Scale bar, 200 µm. **d**, Telomerase activities of NMR-iPSCs. NMR-Fibro, NMR-fibroblasts; human-Fibro, human skin fibroblasts (TIG113); N.C., Heat-inactivated (+) samples; I.C., internal control; P.C., positive control. *n* = 3 clones. **e**, Proliferation of NMR-iPSCs (clones 24 and 27). **f**, Population doubling times (PDT). *n* = 3 clones. **P* < 0.05; N.S., not significant (*t*-test). **g**, qRT-PCR analysis of transgene expression in NMR-iPSCs. NMR-Fibro/4F-5d, NMR-fibroblasts 5 days after the transduction with OSKM. Ms-iPSCs, 20D17. *n* = 4 clones. Results are represented as mean ± SD. **h**, RT-PCR analysis of pluripotency markers in NMR-iPSCs and NMR-fibroblasts. **i**, Immunofluorescence analysis of the expression of pluripotency markers OCT4 and E-cadherin. Hoechst dye (blue), nuclei. Scale bar, 100 µm. **j**, Principal component analysis (PCA) of global gene expression patterns of four NMR-iPSC clones and two NMR-fibroblast lines. **k**, RT-PCR analysis of the expression of pluripotency and differentiation markers in EBs. *MEF2c,* mesoderm; *GATA4*, endoderm; *MAP2* and *MSI1*, ectoderm. U, undifferentiated; D, differentiated. *n* = 3 clones.

Supplementary Figure 2

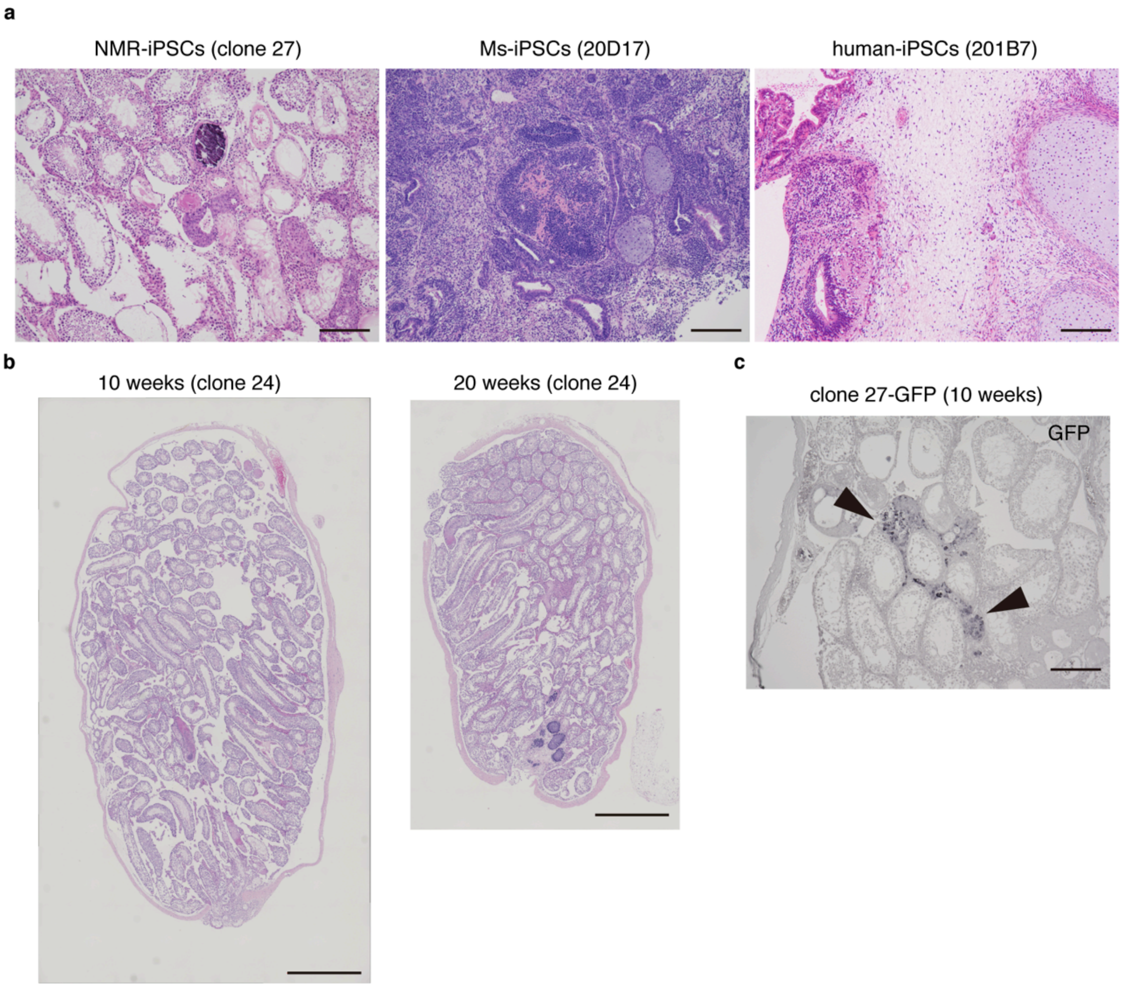

Supplementary Figure 2 | Histopathological analysis of tumours and testes after transplantation of iPSCs. a and **b**, Haematoxylin and eosin staining. Sections of tumours and testes of mice transplanted with NMR-iPSCs (clone 27), Ms-iPSCs (20D17) or human-iPSCs (201B7) (**a**). Scale bar, 200 μm. Testes injected with NMR-iPSCs (clone 24) 10 or 20 weeks after transplantation (**b**). Scale bar, 1 cm. **c**, Immunohistochemical analysis of GFP. Transplanted NMR-iPSCs were lentivirally labelled with GFP. Arrowhead, area of the engrafted site. Scale bar, 200 μm.

Supplementary Figure 3

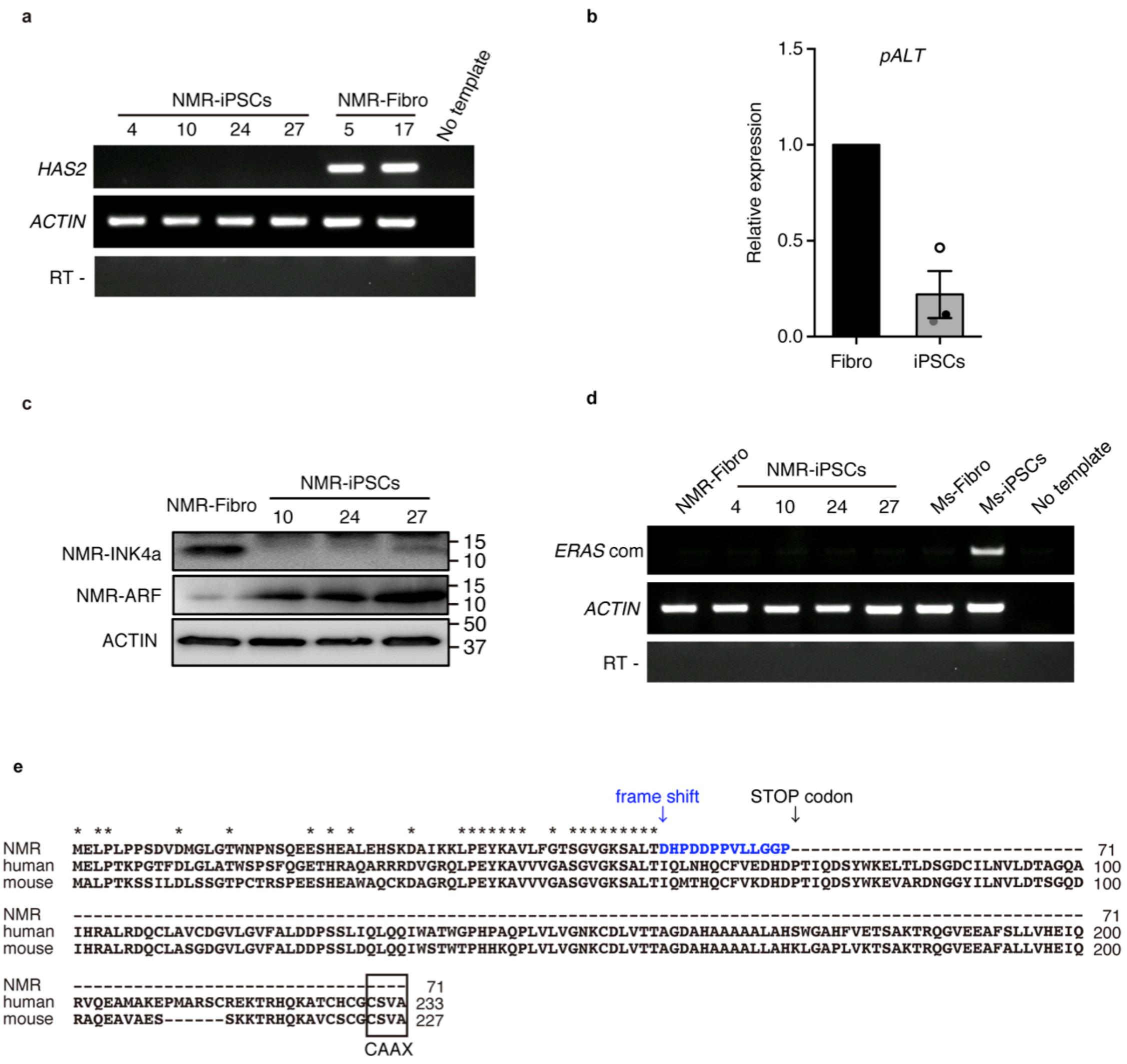

Supplementary Figure 3 | Activation of *ARF* and frameshift mutation of *ERAS* in NMR-iPSCs. a, RT-PCR analysis of *HAS2* expression in NMR-iPSCs. **b**, Expression of *pALT*. Results are presented as mean ± SEM. n = 3 clones. **c**, Western blotting of INK4a and ARF expression in NMR-iPSCs and NMR-fibroblasts. n = 3 clones. **d**, RT-PCR analysis of *ERAS* expression in NMR-iPSCs. Ms-iPSCs (20D17), positive control. *ERAS* com, primer-set designed to amplify the sequence shared by NMR-*ERAS* and *mERas*. **e**, Amino acid sequence comparison among Ms-, human- and NMR-*ERAS* genes. Blue arrow: frameshift mutation. Box: CAAX motif.

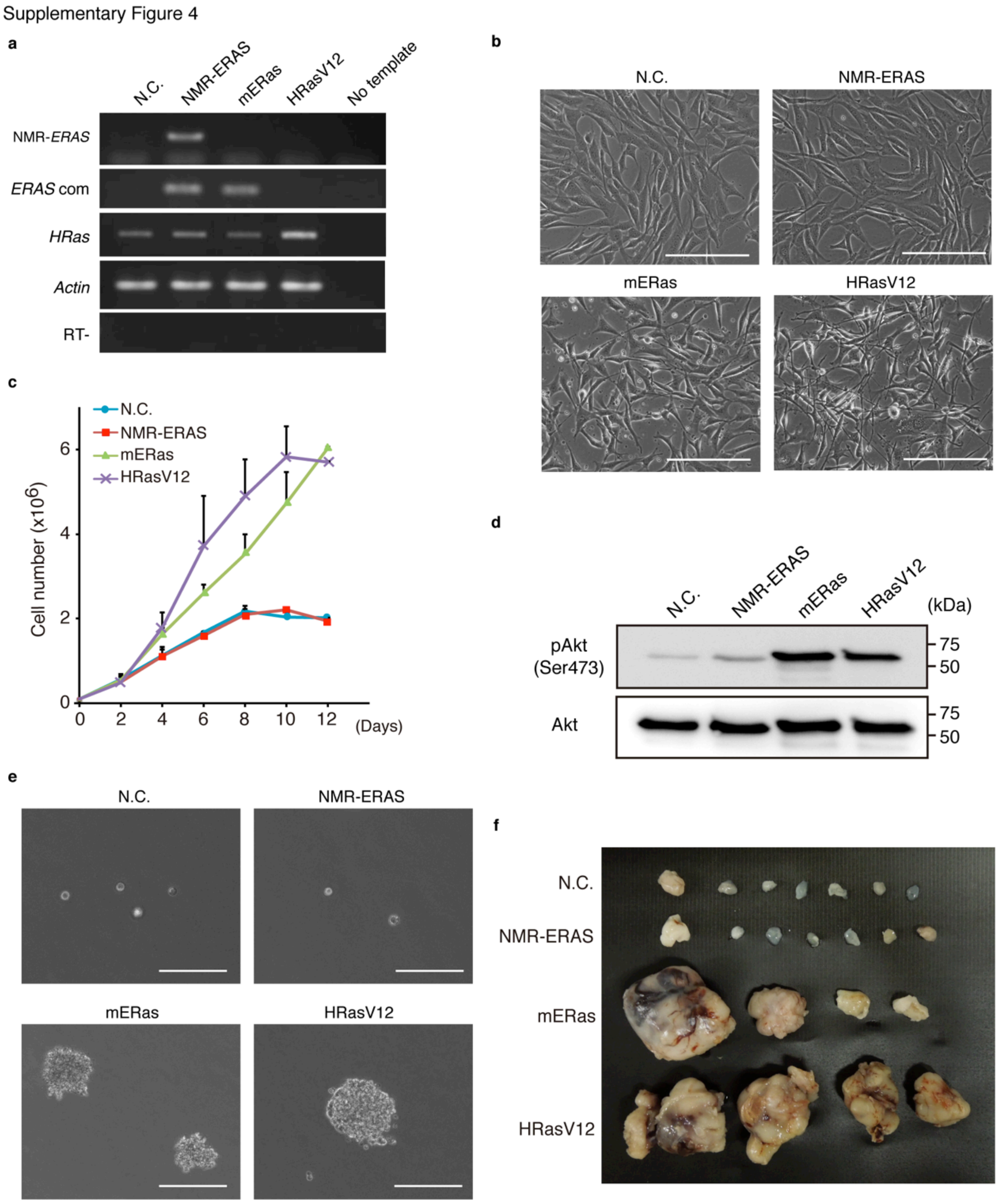

Supplementary Figure 4 | Transforming potential of NMR-ERAS in NIH-3T3 cells. NIH-3T3 cells were infected with lentiviral vectors expressing NMR-ERAS or mERas. HRasV12, positive oncogenic control; N.C., EGFP as negative control. **a**, RT-PCR analysis of transgene expression. **b**, Cell morphology. Scale bar, 200 μm. **c**, Cell proliferation. Cells (1×10^5) were plated on 10 cm dish and counted every other day. Data are represented as mean ± SD. **d**, Western blotting for AKT or phosphorylated-AKT expression. **e**, Soft agar growth assay. Scale bar, 200 μm. **f**, Tumour formation in nude mice. Cells (1×10^6) were subcutaneously injected and tumours were dissected 25 days later.

Supplementary Figure 5

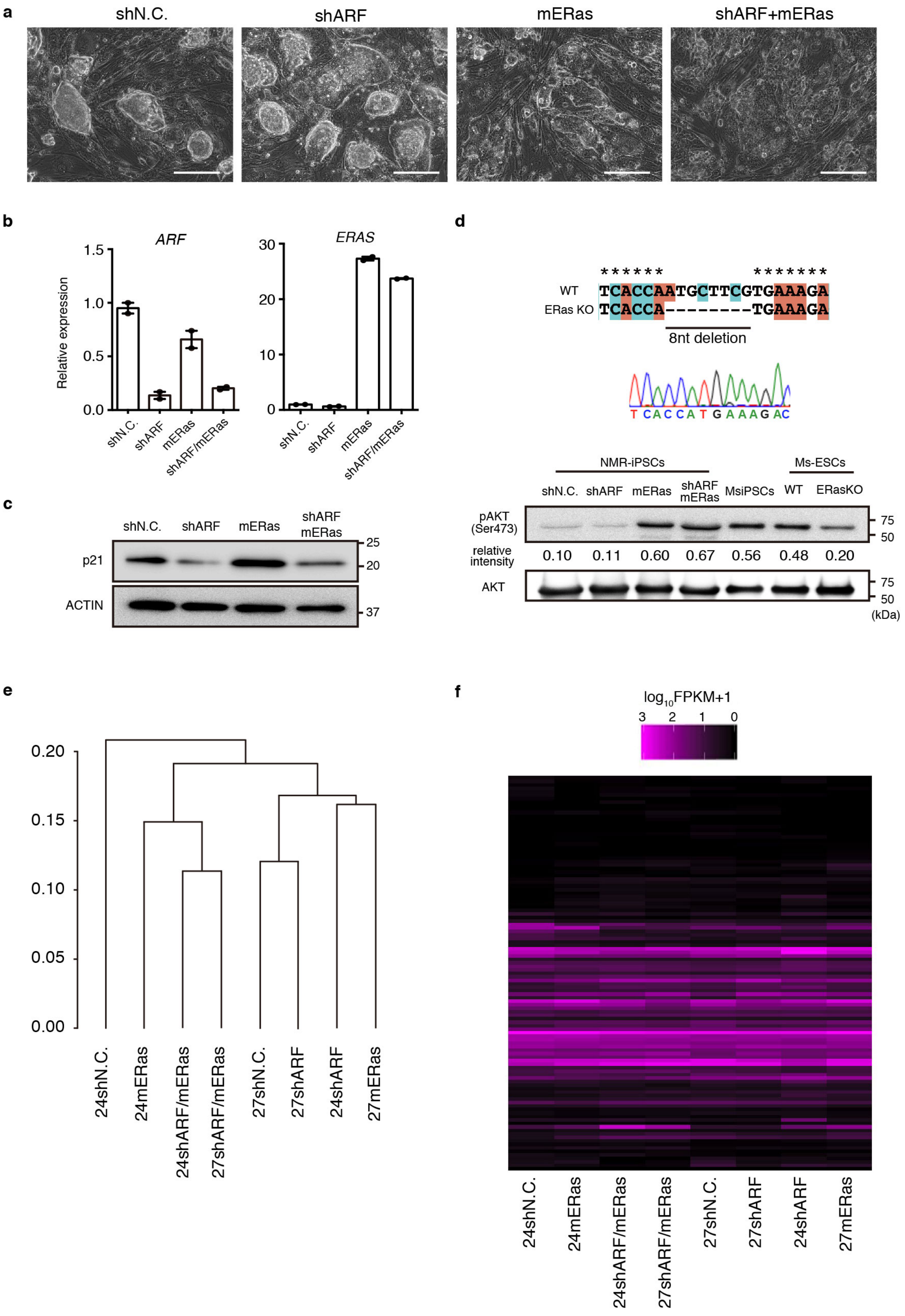

Supplementary Figure 5

g

Gene List

Undifferentiated		Mesoderm	Endoderm	Ectoderm
hDNMT3B	DNMT3L	BMP2	AFP	OTX2
DPPA4	KRT17	BMP4	FOXA1	SLC6A3
ERAS	DPPA2	CDH1	FOXA2	CHAT
FBXO15	TERT	EOMES	KRT19	LMX1B
FGF4	IFITM1	HAND1	SOX17	GFAP
KLF4	NODAL	FABP4	TTR	MAP2
LIN28A	GRB7	VEGFA	GATA4	PAX6
LIN28B	PODXL	MEF2C	PDX1	NES
NANOG	CD9	NKX2-5	KRT8	MSI1
NR0B1	BRIX1	ACTA2	ALB	GFAP
POU5F1	ESRRB	FLT1	PDGFRA	REST
SALL4	RNF17	CDH11	HNF4A	TH
SOX2	NR6A1	RUNX1	HNF1B	SYP
UTF1	NUMB	MESDC2	SERPINA1	MNX1
ZFP42	REST	MYOD1	CPS1	
ZNF296	LIFR	WT1	TAT	
ZSCAN4	T	NPPA	LAMC1	
TDGF1	MYC	HBB	INS	
FGF5	MYCN	RUNX2	FN1	
		COL2A1	PAX4	
			SST	

h

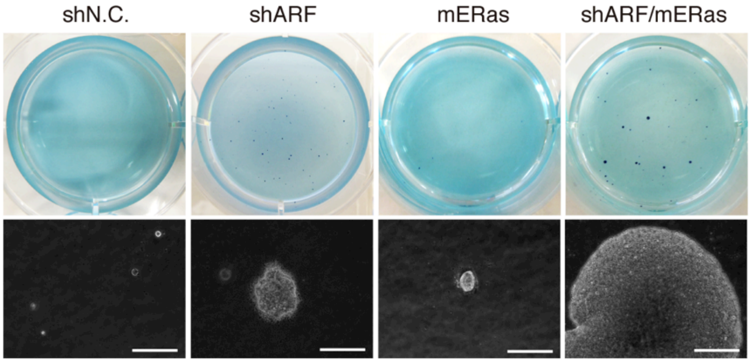

i

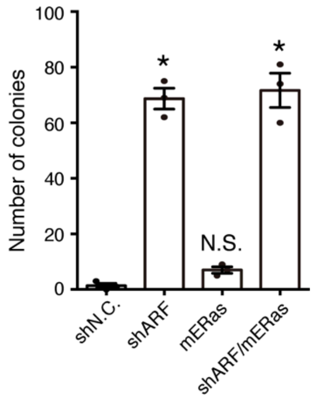

Supplementary Figure 5 | Characterization of NMR-iPSCs expressing mERas and/or shARF. a, Morphology of NMR-iPSCs (clone 24) transduced with mERas and/or shARF. Scale bar, 200 μm. **b**, qRT-PCR analysis of *ARF* and *mERas* expression in NMR-iPSCs. n = 2 clones. Data are represented as mean ± SEM. **c**, Western blotting for p21. **d**, The genomic sequence of *ERas* in *ERas* knock-out Ms-ES cell line (EGR-G101). Western blotting for AKT or phosphorylated-AKT expression in the indicated cell lines. Values indicate the relative intensity of AKT expression and phosphorylation. **e**, Hierarchical clustering analysis. #24, NMR-iPSC clone 24; #27, NMR-iPSC clone 27. **f**, Heat map of the selected markers of undifferentiated and differentiated cells. **g**, Selected genes shown in Supplementary Fig. 5e and f**. h**, Soft agar growth assay of NMR-iPSCs expressing mERas and/or shARF. **i**, Number of colonies. n = 2. Results are presented as mean ± SD for three experimental replicates. *P < 0.05 (one way ANOVA). N.S., not significant. N.C., negative control. Scale bar, 200 μm (**a** and **g**).

Supplementary Figure 6

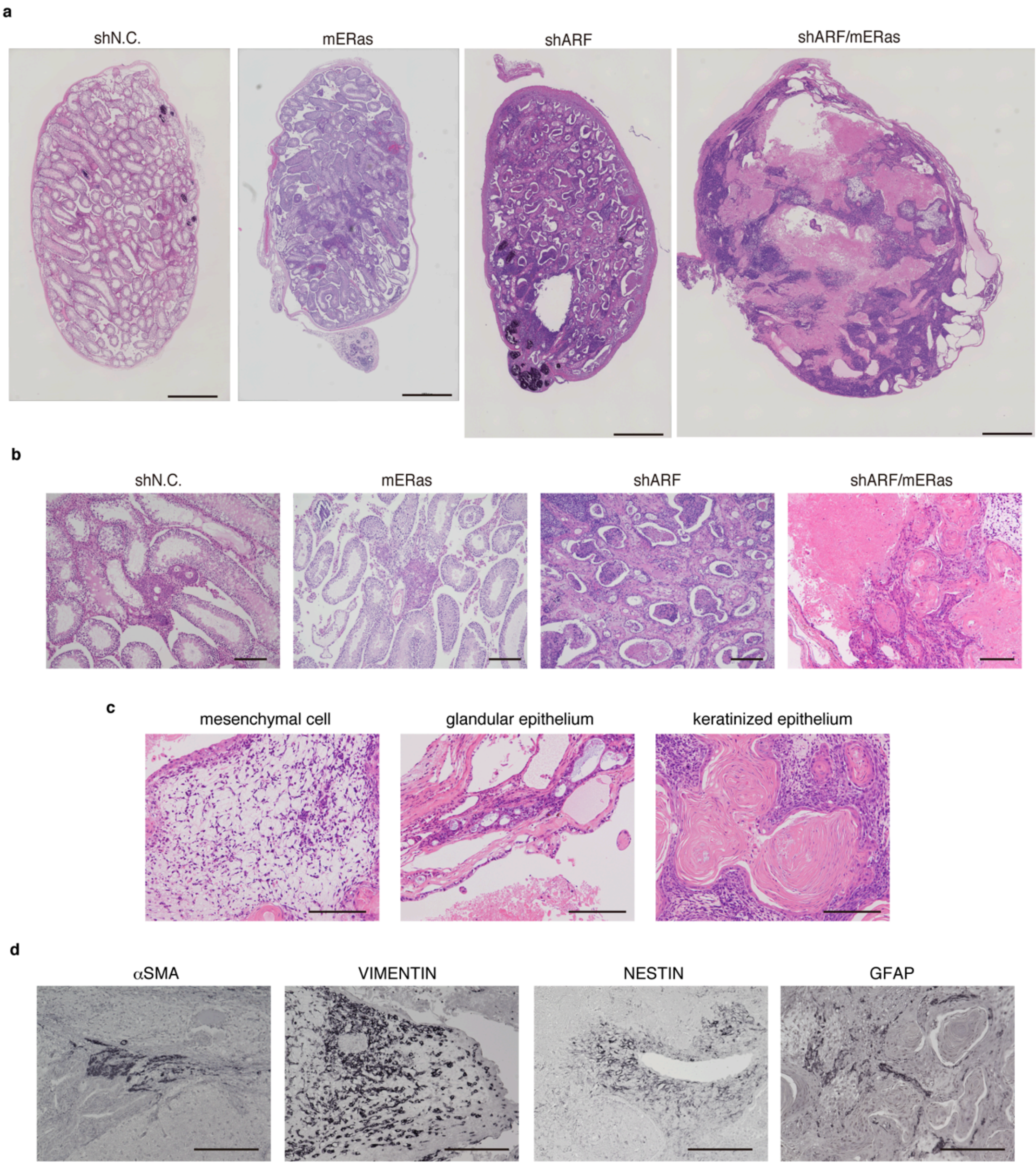

Supplementary Figure 6 | Histopathological analysis of tumours derived from NMR-iPSCs expressing mERas and/or shARF. a, b and **c,** Haematoxylin and eosin staining. Sections of tumours and testes 10 weeks after transplantation of NMR-iPSCs expressing mERas and/or shARF. Testes and tumours (**a**). Scale bar, 1 cm. High magnification (**b**). Scale bar, 200 μm. Representative images of teratomas formed by mERas/shARF-NMR-iPSCs (**c**). Scale bar, 200 μm. **d**, Immunohistochemical analysis of teratomas formed by mERas/shARF-NMR-iPSCs. αSMA, mesoderm; VIMENTIN, mesoderm and parietal endoderm; NESTIN and GFAP, ectoderm. Scale bar, 200 μm.

Supplementary Figure 7

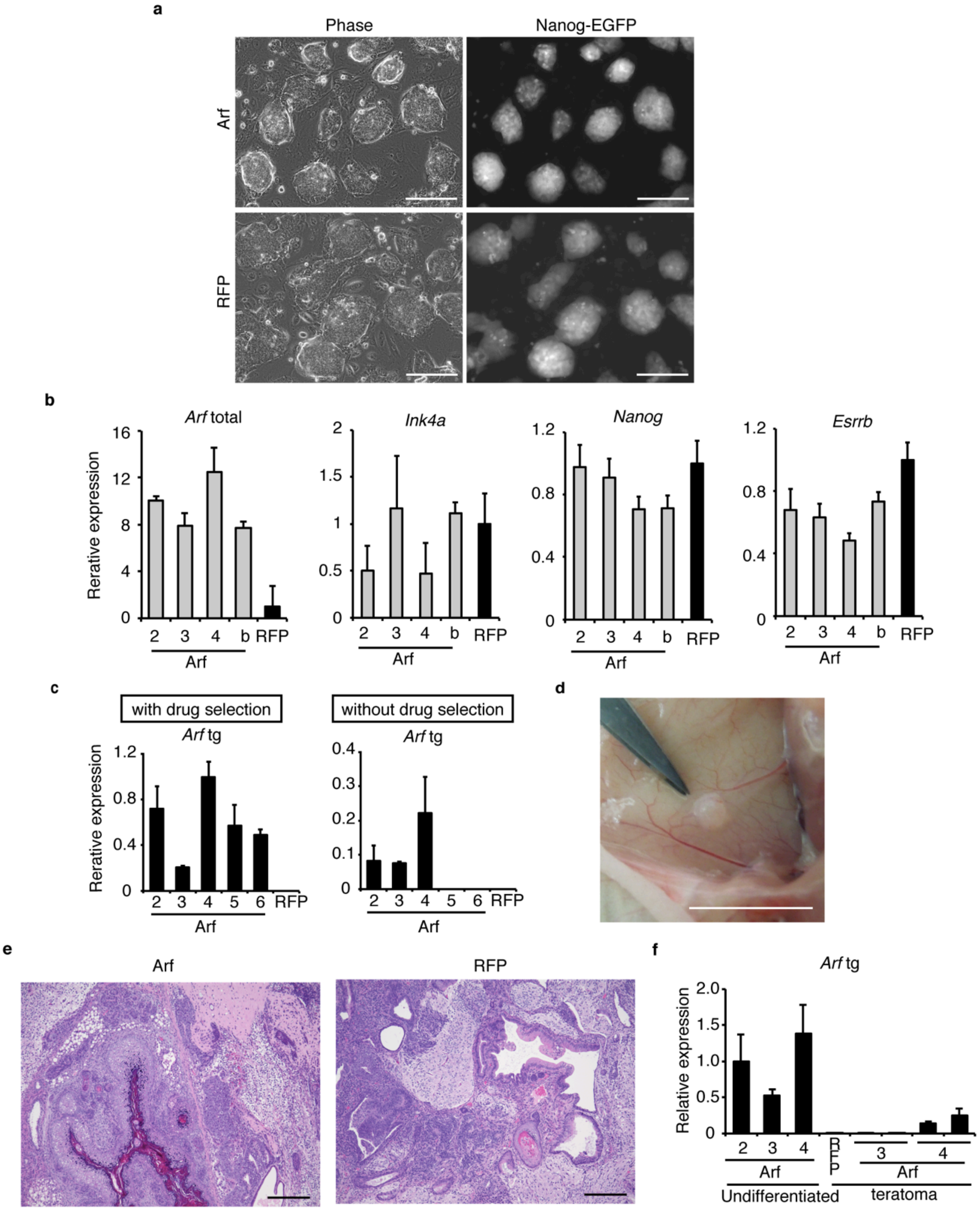

Supplementary Figure 7 | Characterization of Ms-iPSCs expressing Arf. a, Morphology of Ms-iPSCs (20D17) expressing Arf. GFP: Nanog reporter (Nanog-GFP). Scale bar, 200 μm. **b**, qRT-PCR analysis of total *Arf* (endogenous and transgenic), *Ink4a*, *Nanog* and *Esrrb* expression in Arf-Ms-iPSCs. *n* = 3 clones (2, 3 and 4). b, bulk culture. **c**, Expression of the *Arf* transgene (*ARF* tg) in Arf-Ms-iPSC clones with (left panel) or without (right panel) hygromycin selection.

Experimental duplicate. **d**, Small teratomas observed 10 weeks after transplantation of the High-ARF-group clone 3. Scale bar, 1 cm. **e**, Haematoxylin and eosin staining of a teratoma derived from High-ARF-group clone 3 (12 weeks) or control Ms-iPSCs (3 weeks) after transplantation. **f,** qRT-PCR analysis of *Arf* transgene expression in teratomas or undifferentiated iPSCs. Scale bar, 200 μm. Data represent the mean ± SD (**b**, **c** and **f**).

Supplementary Figure 8

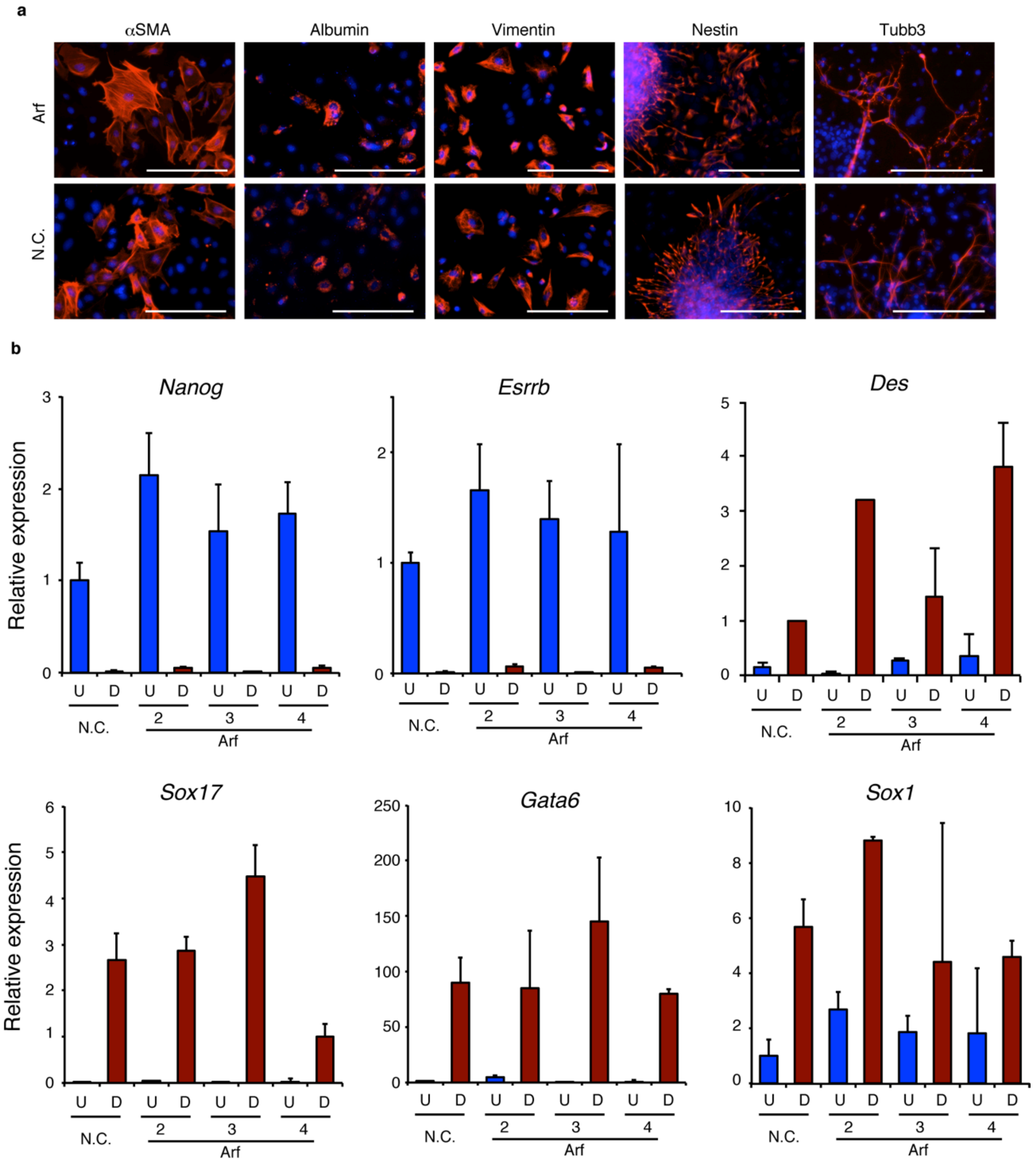

Supplementary Figure 8 | Differentiation potential of Arf-Ms-iPSCs. a, Immunocytochemical analysis of differentiated cells from the High-Arf-group clone 4. mesoderm (αSMA), endoderm (Albumin and Vimentin), ectoderm (Nestin and Tubb3). Scale bar, 200 μm. **b**, qRT-PCR analysis of differentiated cells from Arf-Ms-iPSCs (clone 2, 3, 4). pluripotent marker genes (*Nanog* and *Esrrb*) and differentiation marker genes (*Des*, *Sox17*, *Gata6* and *Sox1*). U, undifferentiated; D, differentiated. Data represent the mean ± SD.

Supplementary Figure 9

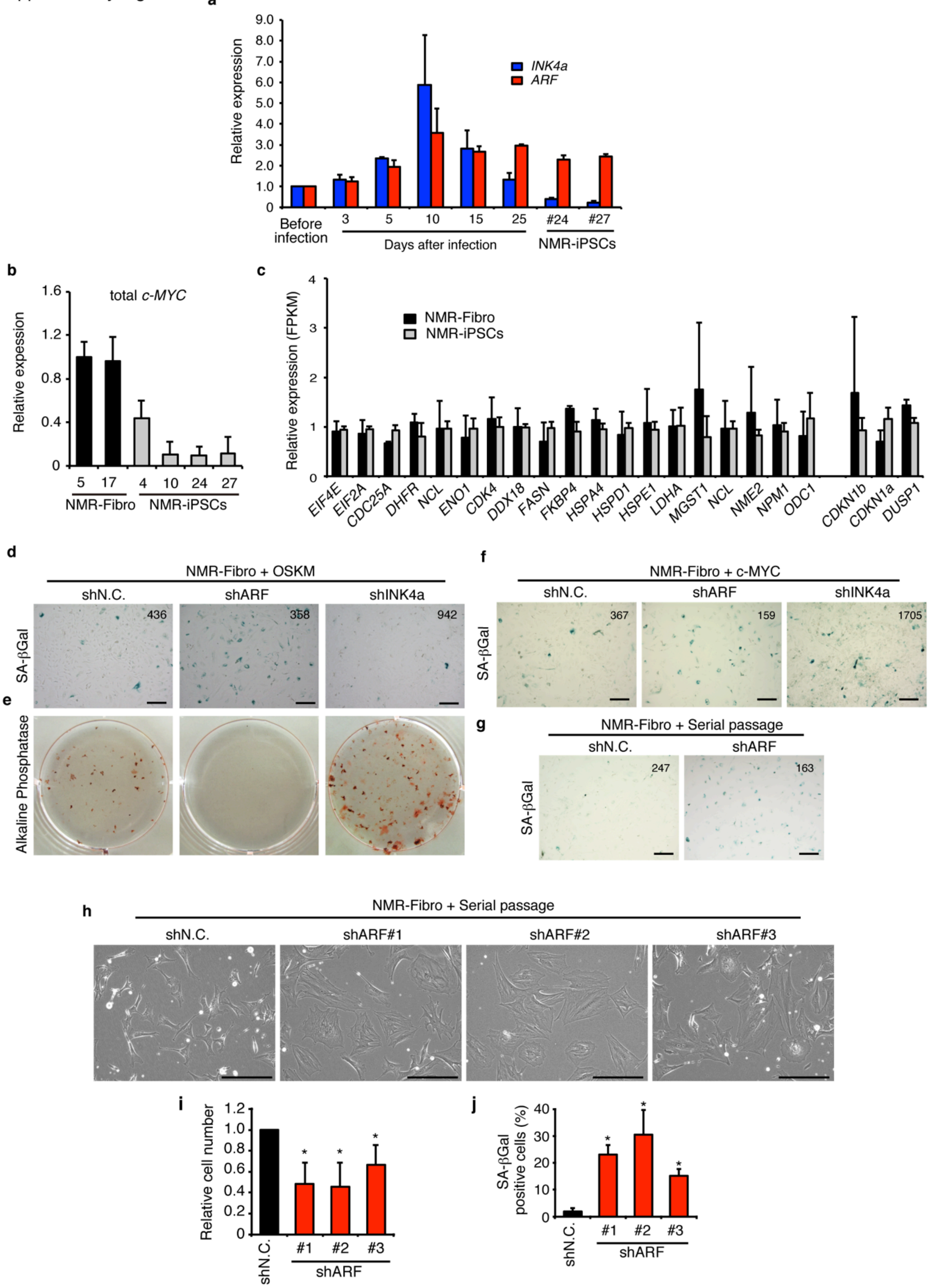

Supplementary Figure 9 | ASIS as a safeguard against reprogramming and oncogenic transformation. a, qRT-PCR analysis of the kinetics of *INK4a* and *ARF* expression during

reprogramming of NMR-fibroblasts. Experimental triplicate. **b**, qRT-PCR analysis of the expression of total *c-MYC* in NMR-iPSCs and parental NMR-fibroblasts. The primers amplified endogenous and transgenic *c-Myc*. n = 4 clones. **c**, RNA-seq of the levels of c-MYC target genes. Nineteen genes on the left, upregulated genes by c-Myc; three on the right, downregulated genes by c-Myc. Y-axis: expression level (FPKM) of NMR-fibroblasts relative to NMR-iPSCs clone 4. **d**, SA-βGal activity of NMR-fibroblasts expressing shARF and/or shINK4a and OSKM 14 days after infection. **e**, AP activity of NMR-fibroblasts expressing shARF or shINK4a and OSKM 37 days after infection. **f**, SA-βGal activity of NMR-fibroblasts expressing shARF or shINK4a and c-MYC. **g**, Transduction of shARF of fibroblasts with derepressed ARF expression induced by serial passage. SA-βGal activity 14 days after transduction. **h, i** and **j**, Reproducibility of the experiment of ASIS in serial passaged NMR fibroblasts using three independent short hairpins RNA against ARF. Cell morphology (**h**). Cell growth (**i**). SA-βGal-positive cells (%) (**j**). Data are represented as mean ± SD. $*P < 0.05$ (t-test). Scale bar, 200 μm. The number in the right upper corner indicates Hoechst-positive cells.

Supplementary Figure 10

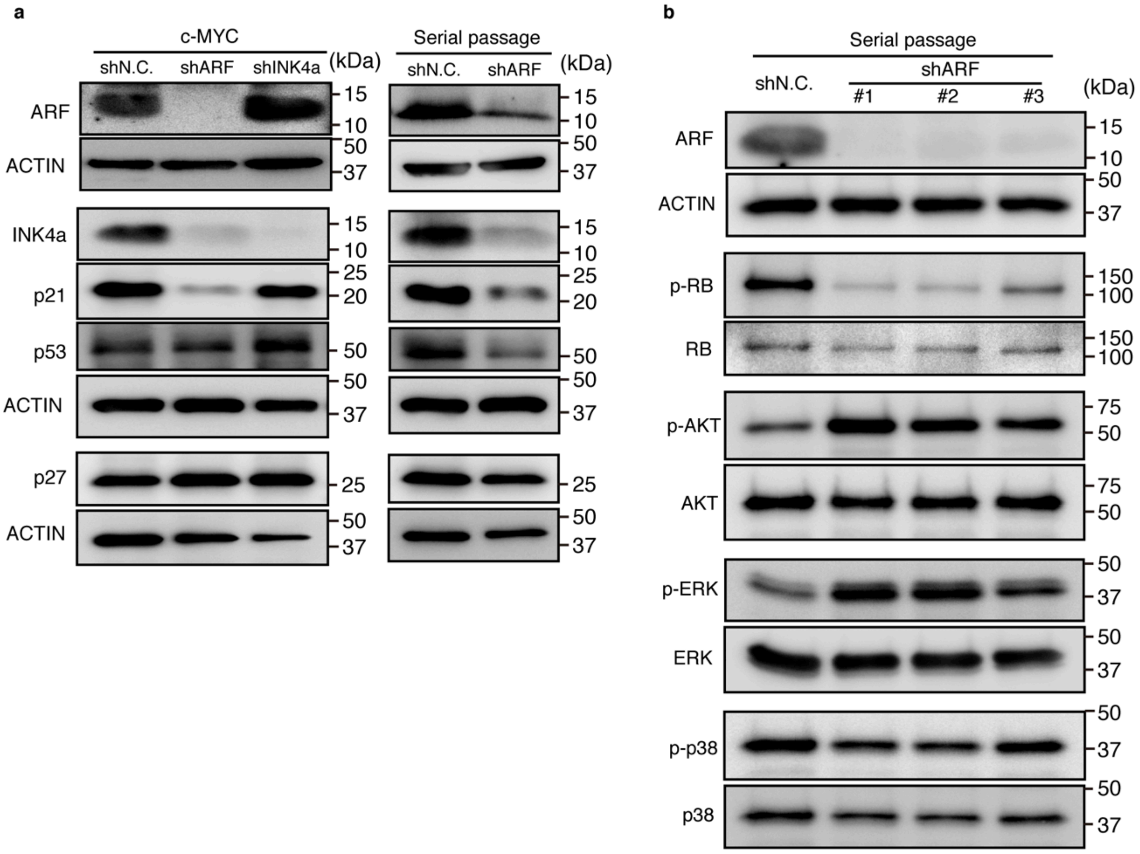

Supplementary Figure 10 | Expression status of senescence-associated genes in NMR fibroblast undergoing ASIS.

a, Western blotting of ARF, INK4a, p21 and p53 expression in stressed NMR-fibroblasts expressing shARF or shINK4a. **b**, Western blotting of RB, AKT and MAPK (ERK and p38) expression in NMR fibroblast undergoing ASIS.

Supplementary Figure 11

Supplementary Fig. 4d pAkt

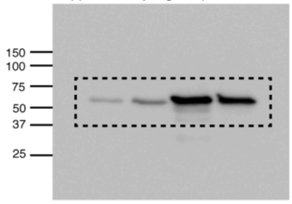

Supplementary Fig. 4d Akt

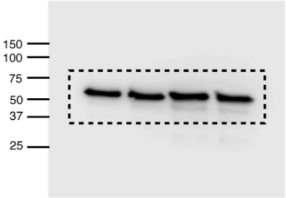

Supplementary Fig. 5c p21

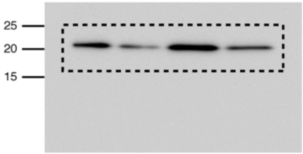

Supplementary Fig. 5c ACTIN

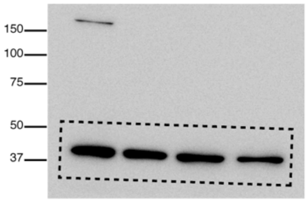

Supplementary Fig. 5d pAKT

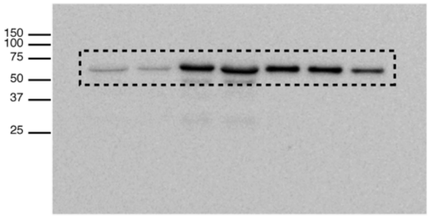

Supplementary Fig. 5d AKT

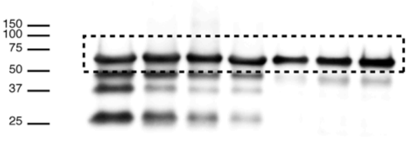

Supplementary Fig. 10a ACTIN

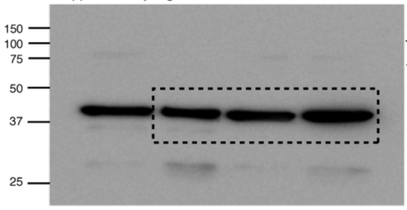

Supplementary Fig. 10a ARF

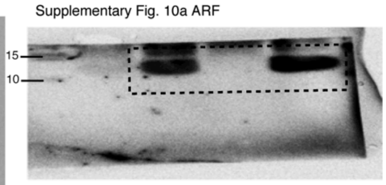

Supplementary Fig. 10a INK4a

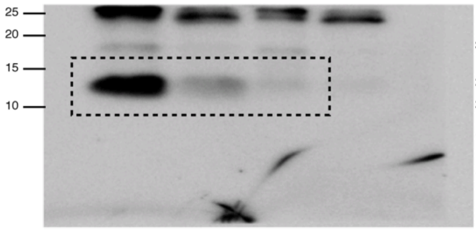

Supplementary Fig. 10a p21

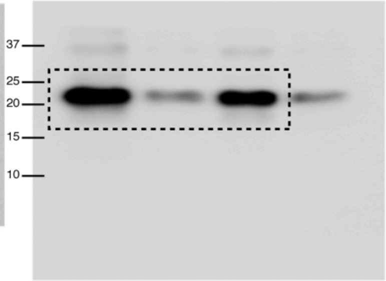

Supplementary Fig. 10a p53

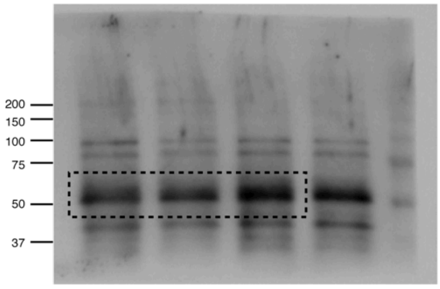

Supplementary Fig. 10a ACTIN

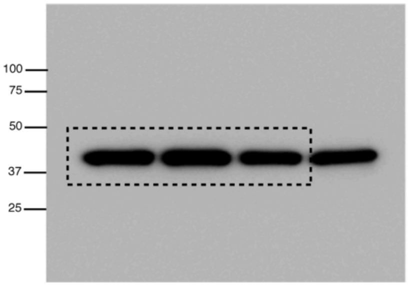

Supplementary Figure 11

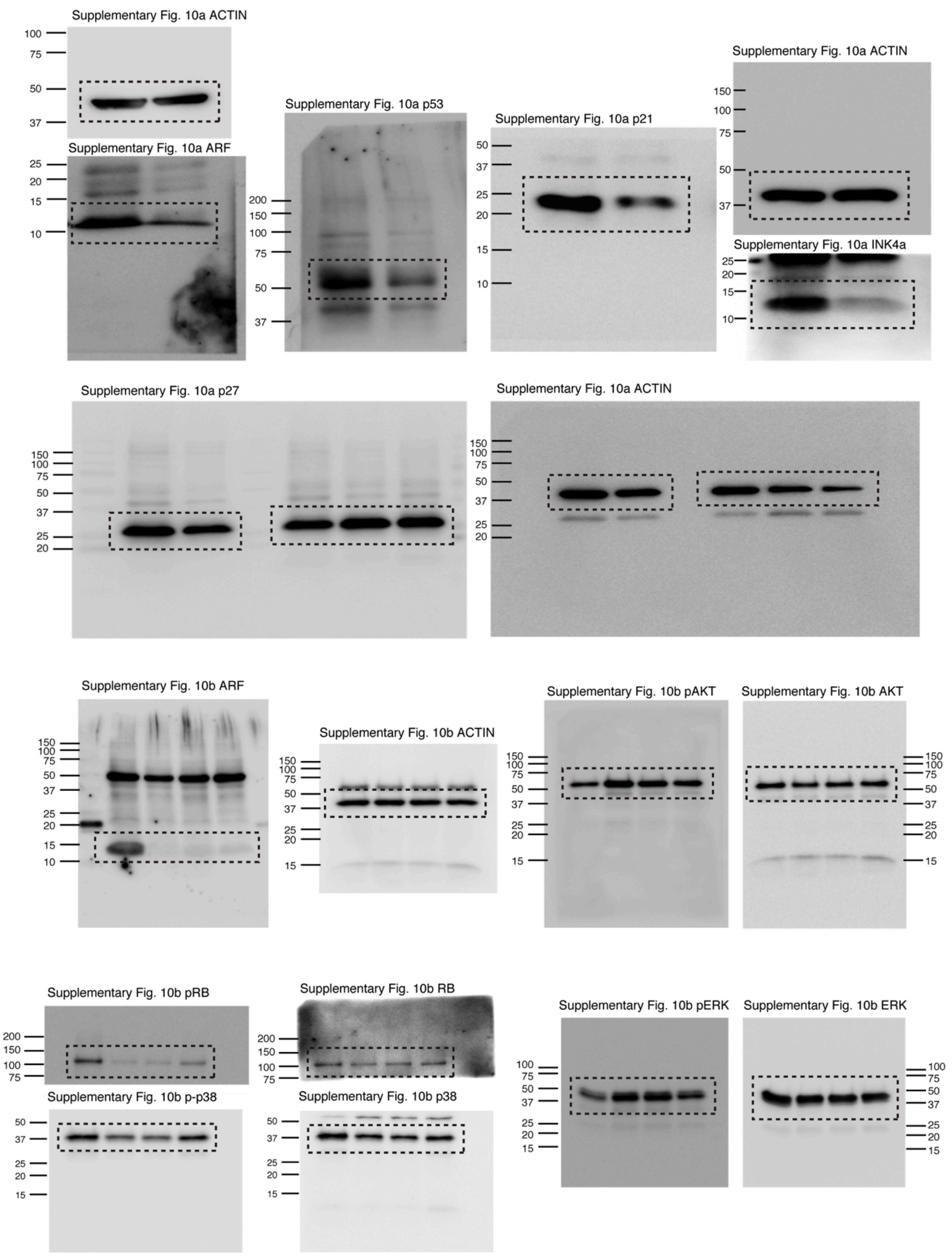

Supplementary Figure 11 | Uncropped scans of western blot results displayed in this study

Supplementary Table 1 | Primers sequences

NMR-INK4a-F	GACCCGAACTGCGCTGACCCT	qRT-PCR for NMR-INK4a
NMR-INK4a-R	CCGCGTCATGCACCGGTAGTGTGA	
NMR-ARF-F	CCATCGTGTGTGCGGGCTTTCGT	qRT-PCR for NMR-ARF
NMR-ARF-R	CTGCTGCCCTCTCCGGTGTCT	
NMR-INK4b-F	AACGCCGTCAATCGCTTCGGGAG	qRT-PCR for NMR-INK4b
NMR-INK4b-R	TCCTCAGCTAGGTCCACGGGCAA	
NMR-CDKN1a-F	ACCTGTCGCTGTCCTGCACCCTTG	qRT-PCR for NMR-p21
NMR-CDKN1a-R	CGTCATGCTGGTCTGCCGCCGTT	
pMXs-Oct4-F	GACTGCCGGATCTAGCTAGTT	qRT-PCR for Oct4 tg
pMXs-Oct4-R	GGGGTGAGAAGGCGAAGTCTGA	
pMXs-Sox2-F	CATGGCCCAGCACTACCAGA	qRT-PCR for Sox2 tg
pMXs-Sox2-R	CCTTACGCGAAATACGGGCAGA	
pMXs-Klf4-F	GCCGACACCAGACTAAGAACC	qRT-PCR for Klf4 tg
pMXs-Klf4-R	GGAGAAGGACGGGAGCAGAG	
pMXs-cMyc-F	GGAATTCGCCCTTCACCATGC	qRT-PCR for c-Myc tg
pMXs-cMyc-R	TGGGAAGCAGCTCGAATTTCT	
NMR-NANOG-F	AAGTACCTCAGCCTGCAGCAGATGC	RT-PCR for NMR-NANOG
NMR-NANOG-R	TTTTCTGCCACCGCTTACATTTCAT	
NMR-FGF4-F	CGTGAGCATCTTTGGAGTGGCCAGC	RT-PCR for NMR-FGF4
NMR-FGF4-R	CAGCCTGGGGAGAAAGTGGGTGAC	
NMR-TERT-F	CCTGCTCAAGCTGGGTCATCACCGTG	RT-PCR for NMR-TERT
NMR-TERT-R	TCAGTCCAGGATGGTCTTGAAGT	
ACTB-F	ACAACGGCTCCGGCATGTGCAA	RT-PCR and qPCR for ACTIN common Ms and NMR
ACTB-R	CATTGTAGAAGGTGTGGTGCCAGA	
NMR-MEF2c-F	GTTTAACACAGCCAGTGCGCTTC	RT-PCR for NMR-MEF2c
NMR-MEF2c-R	CTCTCTGTCGCTGCCGTCGTA	
NMR-GATA4-F	CCCTCCATCCACCCGGTCCTC	RT-PCR for NMR-GATA4
NMR-GATA4-R	ACGCAGTGATTATGTCCCCGTGACT	
NMR-MAP2-F	GATTCTCCTATGCCAAGTCCCT	RT-PCR for NMR-MAP2
NMR-MAP2-R	CTGTTTCTGCCACTTTATCAGGT	
NMR-MSI1-F	AGCTCGACTCCAAAACAATTGACCC	RT-PCR for NMR-MSI1
NMR-MSI1-R	CATCCACCTTCCCGAACTGCT	
NMR-HAS2-F	GAAAAGGGTCCTGGTGAGACGGATGAG	RT-PCR for NMR-HAS2[14]
NMR-HAS2-R	TTCACCATCTCCACAGATGAGGCAGG	
pALT-p15S-F	CAGGAAAAGCCCGGAACTAACTAC	qRT-PCR for NMR-pALT[16]
pALT-p16AS-R	GGTGACAGGGTCAGCGCAGTTCG	
NMR-ERAS-Cl-F	CACCATGGAGCTGCCACTGCCACCTAGT	NMR-ERAS cloning
NMR-ERAS-Cl-R	TCATGGTCCTCCAAGAAGCACT	
ERAS-com-F	CACAGAGCAGCCACAGCTACAC	RT-PCR for Ms and NMR ERAS
ERAS-com-R	GGCAAGGTGTGGAGGAAGCCTT	
NMR-ERAS-F	TTCCCACCTGCTTCTGCCAT	RT-PCR for NMR-ERAS
NMR-ERAS-R	GCTCCCTTCGTGGAAACCTCA	
HRasV12-F	AAGAGTGCGCTGACCATCCAG	RT-PCR for HRasV12
HRasV12-R	TTTGATCTGCTCCCTGTACTGGTG	
Ms-Ink4a-F	GTGTGCATGACGTGCGGG	qRT-PCR for Ms-Ink4a
Ms-Ink4a-R	GCAGTTCGAATCTGCACCGTAG	
Ms-Arf-F	GCTCTGGCTTTCGTGAACATG	qRT-PCR for Ms-Arf
Ms-Arf-R	TCGAATCTGCACCGTAGTTGAG	
Ms-Ink4b-F	AGATCCCAACGCCCTGAAC	qRT-PCR for Ms-Ink4b
Ms-Ink4b-R	CCCATCATCATGACCTGGATT	
Ms-Cdkn1a-F	TCCCGTGGACAGTGAGCAGTTG	qRT-PCR for Ms-p21
Ms-Cdkn1a-R	CGTCTCCGTGACGAAGTCAAAG	
Ms-Nanog-F	TTCTTGCTTACAAGGGTCTGC	qRT-PCR for Ms-Nanog
Ms-Nanog-R	AGAGGAAGGGCGAGGAGA	
Ms-Esrrb-F	CTGCCGATTTCCCCACCTG	qRT-PCR for Ms-Esrrb
Ms-Esrrb-R	TGAGGAACACAAGCTCCCGAT	
Arf-tg-F	CCAAGAGCGGGGACATCAAGACA	qRT-PCR for Arf tg
Arf-tg-R	CCACACCAGCCACCACCTT	
Ms-Des-F	AGCTCTCCCGTGTTCCCTC	qRT-PCR for Ms-Des
Ms-Des-R	CAGCGACCCCAAGCCTCC	
Ms-Sox17-F	GTGGACCGCACGGAATTCGAA	qRT-PCR for Ms-Sox17[45]
Ms-Sox17-R	GCAATAGTAGACCGCTGAGCTA	
Ms-Gata6-F	ACCTTATGGCGTAGAAATGCTGAGGGTG	qRT-PCR for Ms-Gata6
Ms-Gata6-R	CTGAATACTTGAGGTCACTGTTCTCGGG	
Ms-Sox1-F	TGAACGCCTTCATGGTGTGGTC	qRT-PCR for Ms-Sox1[45]
Ms-Sox1-R	GCGCGGCCGGTACTTGTAAT	
cMYC-com-F	TGCTCCACCTCCAGCCTGTACCT	pRT-PCR for Ms and NMR c-MYC
cMYC-com-R	CCTCATCCTCTTGCTCTCCTTCAG	

Supplementary Table 2 | shRNA sequences

Target gene	Sequence
NMR-ARF #1	5'-GGGCTTTCGTGGTGCAGATCC -3'
NMR-ARF #2	5'-GCGGGCTTTCGTGGTGCAGAT-3'
NMR-ARF #3	5'-GGCCCTCTTGCTGATGCTAGT-3'
NMR-INK4a	5'-GGTCCAGGAGGTACGCGAGCT-3'
